# SB225002 Induces Cell Death and Cell Cycle Arrest in Acute Lymphoblastic Leukemia Cells through the Activation of *GLIPR1*

**DOI:** 10.1371/journal.pone.0134783

**Published:** 2015-08-24

**Authors:** Jaíra Ferreira de Vasconcellos, Angelo Brunelli Albertoni Laranjeira, Paulo C. Leal, Manoj K. Bhasin, Priscila Pini Zenatti, Ricardo J. Nunes, Rosendo A. Yunes, Alexandre E. Nowill, Towia A. Libermann, Luiz Fernando Zerbini, José Andrés Yunes

**Affiliations:** 1 Centro Infantil Boldrini, Campinas, SP, Brazil; 2 Department of Medical Genetics, Faculty of Medical Sciences, University of Campinas, Campinas, SP, Brazil; 3 BIDMC Genomics, Proteomics, Bioinformatics and Systems Biology Center, Beth Israel Deaconess Medical Center and Harvard Medical School, Boston, Massachusetts, United States of America; 4 Department of Chemistry, Santa Catarina Federal University, Florianopólis, SC, Brazil; 5 Centro Integrado de Pesquisas Oncohematológicas da Infancia, University of Campinas, Campinas, SP, Brazil; 6 Cancer Genomics Group, International Center for Genetic Engineering and Biotechnology and Medical Biochemistry Division, University of Cape Town, Cape Town, South Africa; Virginia Commonwealth University, UNITED STATES

## Abstract

Acute Lymphoblastic Leukemia (ALL) is the most frequent childhood malignancy. In the effort to find new anti-leukemic agents, we evaluated the small drug SB225002 (N-(2-hydroxy-4-nitrophenyl)-N’-(2-bromophenyl)urea). Although initially described as a selective antagonist of CXCR2, later studies have identified other cellular targets for SB225002, with potential medicinal use in cancer. We found that SB225002 has a significant pro-apoptotic effect against both B- and T-ALL cell lines. Cell cycle analysis demonstrated that treatment with SB225002 induces G2-M cell cycle arrest. Transcriptional profiling revealed that SB225002-mediated apoptosis triggered a transcriptional program typical of tubulin binding agents. Network analysis revealed the activation of genes linked to the *JUN* and *p53* pathways and inhibition of genes linked to the *TNF* pathway. Early cellular effects activated by SB225002 included the up-regulation of *GLIPR1*, a p53-target gene shown to have pro-apoptotic activities in prostate and bladder cancer. Silencing of *GLIPR1* in B- and T-ALL cell lines resulted in increased resistance to SB225002. Although SB225002 promoted ROS increase in ALL cells, antioxidant N-Acetyl Cysteine pre-treatment only modestly attenuated cell death, implying that the pro-apoptotic effects of SB225002 are not exclusively mediated by ROS. Moreover, *GLIPR1* silencing resulted in increased ROS levels both in untreated and SB225002-treated cells. In conclusion, SB225002 induces cell cycle arrest and apoptosis in different B- and T-ALL cell lines. Inhibition of tubulin function with concurrent activation of the *p53* pathway, in particular, its downstream target *GLIPR1*, seems to underlie the anti-leukemic effect of SB225002.

## Introduction

Acute lymphoblastic leukemia (ALL) is the most common cancer in childhood. We previously reported that the leukemic bone marrow (BM) microenvironment has increased levels of chemokine (C-C motif) ligand 2 (CCL2) and interleukin-8 (IL-8), and that these chemokines have a positive impact on BM mesenchymal stem cells, but no direct effect on ALL cells [1]. In our efforts to evaluate the function of the IL-8 receptor CXCR2 in ALL cells, we made use of SB225002 (N-(2-hydroxy-4-nitrophenyl)-N’-(2-bromophenyl)urea), a drug initially described as a CXCR2 antagonist [2]. Although the CXCR2 receptor was found to be non-functional in ALL [1], B- and T-ALL cell lines were sensitive to SB225002.

We learned with interest throughout the course of this project that SB225002 is not a specific inhibitor of CXCR2 as it was stated in its first description [2]. Now, SB225002 is known to have many cellular targets and effects, including the inhibition of microtubule polymerization, hyperphosphorylation of Bcl2 and BclxL, prometaphase cell cycle arrest, promotion of mitotic catastrophe and inhibition of gamma-secretase [3–5]; amelioration of acute experimental colitis *in vivo* [6]; management of both acute and chronic pain *in vivo* [7]; angiogenesis inhibition *in vivo* [8]; among others.

Notwithstanding, SB225002 has potentially interesting anti-cancer effects, which have been previously reported in esophageal cancer [9], pancreatic cancer with activated K-Ras [10], breast cancer [11], oral squamous cell carcinoma [12], ovarian cancer [5], lung adenocarcinoma [13], nasopharyngeal carcinoma [14], clear cell renal cell carcinoma [15], intrahepatic cholangiocellular carcinoma [16] and metastatic breast cancer cells [17]. In this manuscript we address for the first time, SB225002’s anti-leukemic effects against acute lymphoblastic leukemia.

## Materials and Methods

### Ethics Statement

Institutional Review Board approval for the animal study was obtained from the Ethics Commission for Animal Use from the Institute of Biology at the University of Campinas (CEUA/UNICAMP, protocol 3624–1). The use of a patient ALL sample in this study was approved by the Centro Infantil Boldrini Ethics Committee (CAAE 0004.0.144.000–05). The patient-derived sample corresponded to frozen patient-derived xenograft cells, whose primary tumors were obtained in the early 1990s. The ethics committee has exceptionally waived the informed consent for those leukemia samples collected prior to the start of the study because it could not be obtained due to death or loss to follow-up.

### Reagents

SB225002 was synthesized following the method described by White et al. [2] or was commercially obtained from Calbiochem (San Diego, CA, USA), dissolved in dimethyl sulfoxide (DMSO) from Sigma-Aldrich (St. Louis, MO, USA) and cells were treated in RPMI-1640 medium in different timepoints. The final concentrations of SB225002 ranged from 1.5625 to 100 μM. For the controls, cells were treated with an equal amount of DMSO (Sigma-Aldrich), which was at maximum 0.1% final concentration. N-Acetyl Cysteine (Sigma-Aldrich) was diluted in water and used at a final concentration of 10 mM.

### Cell Culture

The Jurkat cell line was kindly provided by Dr. George C. Tsokos, Beth Israel Deaconess Medical Center, Boston, MA, USA [18]; the REH cell line was kindly provided by Dr. Leslie E. Silberstein, Children’s Hospital Boston, Boston, MA, USA [19]; the cell lines 697 and RS4;11 were kindly provided by Dr. Sheila A. Shurtleff, St. Jude Children’s Research Hospital, Memphis, TN, USA [20, 21]; the cell line TALL-1 was kindly provided by Dr. João Barata, Instituto de Medicina Molecular, Faculdade de Medicina da Universidade de Lisboa, Lisboa, Portugal [22]; and the cell lines Nalm-6, CEM and Molt-4 were kindly provided by Dr. Angelo Cardoso, Indiana University School of Medicine, I.U. Simon Cancer Center, Indianapolis, IN, USA [21, 23]. Cell lines were grown in RPMI-1640 medium (Fisher/Thermo Scientific, Pittsburgh, PA, USA) and supplemented with 10% fetal bovine serum, 50 U/ml penicillin and 50 μg/ml of streptomycin (all GIBCO, Carlsbad, CA, USA). Post-ficoll lymphocytes obtained from normal healthy volunteers were grown in RPMI-1640 medium supplemented with 10% fetal bovine serum and stimulated with phytohemagglutinin (PHA) for 3 days. Cells were maintained in a 5% CO_2_-humidified incubator at 37°C.

### Quantitative PCR

Total RNA was extracted using QIAshreder (Qiagen, Valencia CA, USA) followed by total RNA isolation using the RNeasy Mini Kit (Qiagen). cDNAs were generated from 3 μg of total RNA using Ready-to-Go You-prime First-Strand Beads (GE Healthcare, Piscataway, NJ, USA). Amplifications of 0.1 μg cDNA were carried out using SYBR Green I-based real-time PCR on the LightCycler 480 Real-Time PCR System (Roche Applied Science, Indianapolis, IN, USA). All PCR mixtures contained: PCR buffer (final concentration 10 mM Tris-HCl at pH 9.0, 50 mM KCl and 2 mM MgCl_2_), 250 μM dNTP’s (Roche), 5 pmol of each PCR primer, 0.5X SYBR Green I (Molecular Probes, Carlsbad, CA, USA), and 1U Taq DNA polymerase (Promega, Madison, WI, USA) with 2 μl cDNA in a 25 μl final volume reaction mix. Samples were loaded into wells of Multiwell 96-well microplates. After an initial denaturation step of 3 min at 95°C, conditions for cycling were 40 cycles of 30 sec at 95°C, 30 sec at 56°C, 1 min at 72°C, 1 cycle of melting curves at 95°C for 15 sec, 65°C for 2 min, and 97°C continuous and a final cooling step at 10°C for 30 sec. A standard melting-curve cycle was used to check the quality of amplification, such as no primer dimer being formed during PCR. Expression values were normalized with respect to human *GAPDH* or *ABL*. For each run, serial dilutions of human *GAPDH* plasmids were used as standards for quantitative measurement of the amount of amplified DNA. All samples were run in duplicate and the data were presented as ratio of gene/*GAPDH* or gene/*ABL*. Primer sequences were as follows: *BACH2* sense 5’-GAAAACGATGCTGCCATTTT-3’; antisense 5’-TTGGTGCACACTTCTGCTTC-3’; *CX3CR1* sense 5’-GACGGTTGCATTTAGCCATT-3’; antisense 5’-TGCTCAGAACACTTCCATGC-3’; *GLIPR1* sense 5’-AGCTGCACCCAAACTTCACT-3’; antisense 5’-ATCTGCCCAAACAACCTGAG-3’; *c-JUN* sense 5’-CCCCAAGATCCTGAAACAGA-3’; antisense 5’-CCGTTGCTGGACTGGATTAT-3’; *GAPDH* sense 5’-CAAAGTTGTCATGGATGACC-3’; antisense 5’-CCATGGAGAAGGCTGGGG-3’; *ABL* sense 5’-TGGAGATAACACTCTAAGCATAACTAAAGGT-3’ and antisense 5’-GATGTAGTTGCTTGGGACCCA-3’.

### Western Blot analysis

Treated and control whole cell lysates were prepared in lysis buffer (Cell Signaling, Danvers, MA, USA). Eighty to one-hundred μg of protein were electrophoresed in a 10% SDS-polyacrylamide gel (Bio-Rad, Hercules, CA, USA). Proteins were electro-blotted onto PVDF membrane in a 50 mM Tris-base, 20% methanol, and 40 mM glycine electrophoresis buffer. Membranes were incubated in 5% non-fat dry milk in PBST (Phosphate 100 mM, KCl 27 mM, NaCl 1.37 M pH 7.4 after 1X dilution; 0.2% Tween-20) for 1 h. Blots were probed with primary antibody overnight at 4°C in 2% BSA in PBST, and then incubated with a horseradish peroxidase-conjugated secondary antibody (Cell Signaling) in 5% dry milk in PBST for 1 h at room temperature. Bound antibodies were detected by Super Signal West Pico Chemiluminescent Substrate detection reagent (Pierce/Thermo Scientific, Rockford, IL, USA) and visualized by autoradiography. The primary antibodies used for Western blot analysis were: anti-GLIPR1 (Novus Biologicals, Littleton, CO, USA), p-AKT Ser473 (Cell Signaling), p-GSK3beta Ser9 (Cell Signaling), p-PDK1 Ser241 (Cell Signaling), anti-CX3R1, GAPDH and β-actin (all from Santa Cruz Biotechnology, Santa Cruz, CA, USA).

### Microarray analysis

Total RNA was obtained from Jurkat cells treated with 12.5 μM SB225002 or 0.1% DMSO for 6 h and 9 h using QIAshredder (Qiagen) and RNeasy Mini Kit (Qiagen). Treatments were performed in duplicates. Antisense biotinylated cRNA was prepared on the Affymetrix GeneChip Array Station using the GeneChip HT One-Cycle cDNA Synthesis and Gene Chip HT IVT Labeling kits (Affymetrix, Santa Clara, CA, USA). Biotinylated cRNAs were hybridized to the Affymetrix HAT HG-U133A and HAT HG-U133B Array Plate. Array washing and staining were performed on the GeneChip GCAS Array Station following a robotic protocol according to the manufacturer’s instructions (Affymetrix). Arrays were scanned on the GeneChip HT scanner (Affymetrix). Scanned image output files were visually examined for major chip defects and hybridization artifacts and then analyzed with Affymetrix GeneChip Microarray Analysis Suite 5.0 (MAS5) software (Affymetrix). All high-quality arrays were analyzed using the Probe Logarithmic Intensity Error (PLIER) algorithm. Genes were considered to be differentially expressed in a given group, if the 90% lower confidence bound (LCB) of the fold change (FC) between the two groups was above 1.2 [24]. Microarray data along with information from this study have been deposited in the NIH Gene Expression Omnibus database at www.ncbi.nlm.nih.gov/geo under the accession number GSE71212. To understand the biological mechanisms affected by the transcripts that were counter-regulated by the treatment, interactive networks, pathways, and functions analysis was performed using the commercial Systems Biology oriented package Ingenuity Pathways Analysis (IPA 4.0, www.ingenuity.com, QIAGEN, Redwood City, CA). Furthermore, to identify compounds that may have similar or opposite effects compared to SB225002, the differentially expressed genes, after removal of non-HG-U133A probe sets, were used to query the Connectivity Map database build 02 (www.broadinstitute.org/cmap). Details of the Connectivity Map dataset and analytics have been previously described [25].

### Cell cycle analysis

Cells were treated with DMSO [0.1%] or SB225002 with the following concentrations: REH and RS4;11 [10 μM]; Jurkat and TALL-1 [3.125 μM]. After 24 h of treatment, cells were centrifuged and fixed in cold 70% ethanol, washed twice with PBS and stained with 1 ml of propidium iodide solution (50 μg/ml propidium iodide, 3.8 mM sodium citrate in PBS) supplemented with 50 μl RNase A (50 mg/ml) for 1 h at room temperature and analyzed with a FACSCanto cell cytometer (Becton Dickinson, Franklin Lakes, NJ, USA). At least 20,000 cells were collected and the cell cycle profiles were calculated using the BDFACSDiva (Becton Dickinson).

### Apoptosis analysis

B-ALL (REH and RS4;11) and T-ALL (Jurkat and TALL-1) cells were treated with DMSO [0.1%] or SB225002 [5 or 10 μM] for 24 h. Untreated sh.scramble and sh.*GLIPR1* B- and T-ALL cells were analyzed at the timepoints 0 h, 24 h and 48 h. Cells were then washed once with PBS and labeled with annexin-V-FITC and propidium iodide-PE (TACS Annexin V-FITC Apoptosis Detection Kit, R&D Systems, Minneapolis, MN, USA) for 15 min at room temperature. Cells were analyzed with a FACSCanto flow cytometer (Becton Dickinson) using the BDFACSDiva Software (Becton Dickinson).

### Reactive oxygen species analysis

B-ALL (REH and RS4;11), T-ALL (Jurkat and TALL-1) and PHA-stimulated normal lymphocytes were treated with DMSO [0.1%] or SB225002 [5 μM and 10 μM]. Similar experiments were also carried out with the *GLIPR1* knockdown and scramble (negative control sh.RNA) cell lines. Treatments were performed for 24 h. For experiments using N-Acetyl Cysteine (NAC), media was buffered with HEPES 20 mmol/L, cells were pre-incubated with NAC [10 mM] for 3h and then treated with SB225002 [5 or 10 μM] for 6 or 24 h (for ROS analysis) and 48 h (for MTT analysis). Cells were washed twice with PBS and stained with 20 μM of 2',7'-dichlorodihydrofluorescein diacetate (H_2_DCFDA, excitation/emission wavelength: 492–495/517–527 nm, Invitrogen) for 30 min at 37°C. Analysis was performed after the acquisition of 50,000 events on a BD FACSCanto flow cytometer (Becton Dickinson).

### Proliferation assays and cell viability assays

Proliferation assay was performed in 96-well micro-titer plates containing 10,000 ALL cells *per* well. Viable cells were counted on BD FACSCanto flow cytometer at different time points. Cell viability assays were performed in 96-well micro-titer plates containing 30,000 cells *per* well for the ALL cells lines and 200,000 cells *per* well for the PHA-stimulated normal lymphocytes using the MTT reagent (Sigma-Aldrich). The formazan dye formed by the viable cells was quantified by measuring the absorbance of the dye solution at 590 nM.

### shRNAs lentiviral vectors

The lentiviruses encoding shRNA sequences against the *GLIPR1* gene were obtained from Sigma-Aldrich (SHCLNV-NM_006851). Clones TRCN0000123175 and TRCN0000123176 (Sigma-Aldrich) were used for screening, and clone TRCN0000123176 chosen for subsequent experiments. As negative control, cells were transduced with the MISSION Non-Target shRNA Control Vector (Sigma-Aldrich). After 72 h, infected ALL cell lines REH, RS4;11, Jurkat and TALL-1 were selected with 1.5 μg/mL (RS4;11) or 2.5 μg/mL (REH, Jurkat and TALL-1) puromycin during 10 days. Bulk cells, cultured 1 week without puromycin, were used for experiments.

### Xenograft model

Patient-derived xenograft ALL cells were thawed, washed with PBS and 1x10^7^ cells were injected in NOD/SCID (NOD.CB17-Prkdcscid/J) mice (The Jackson Laboratory, Bar Harbor, ME, USA) by the tail vein. ALL engraftment was monitored as previously described [26] and outlined below. Successfully engrafted mice were sacrificed, ALL cells were collected from spleen and liver and 1x10^7^ cells were immediately injected by the tail vein in eight secondary non-irradiated recipient mice for the subsequent experiments. Animals were monitored every 7 days for ALL engraftment as follows: blood was collected by retro-orbital bleeding into EDTA containing tube, mononuclear cells were isolated by ficoll centrifugation and the presence and quantity of ALL cells was analyzed by flow cytometry in a FACSCanto II equipment (Becton Dickinson, Franklin Lakes, NJ), using anti-hCD45-PE (clone HI30, BD Pharmingen, San Diego, CA or EXBIO, Prague, Czech Republic) and anti-mCD45-FITC (clone 30F-11, BD Pharmingen). When human CD45(+) cells reached ≥ 0.5% of peripheral blood cells in half of the animals, mice were randomly distributed into the different treatment groups (n = 4/each group). Mice were treated intraperitoneally with 10 mg/Kg of SB225002 or vehicle once a day, 5 days a week, for 4 weeks. Kaplan-Meier survival curves were compared using the Log-rank test.

## Results

### SB225002 inhibited the proliferation of ALL cell lines at micromolar concentrations

**Fig 1:**
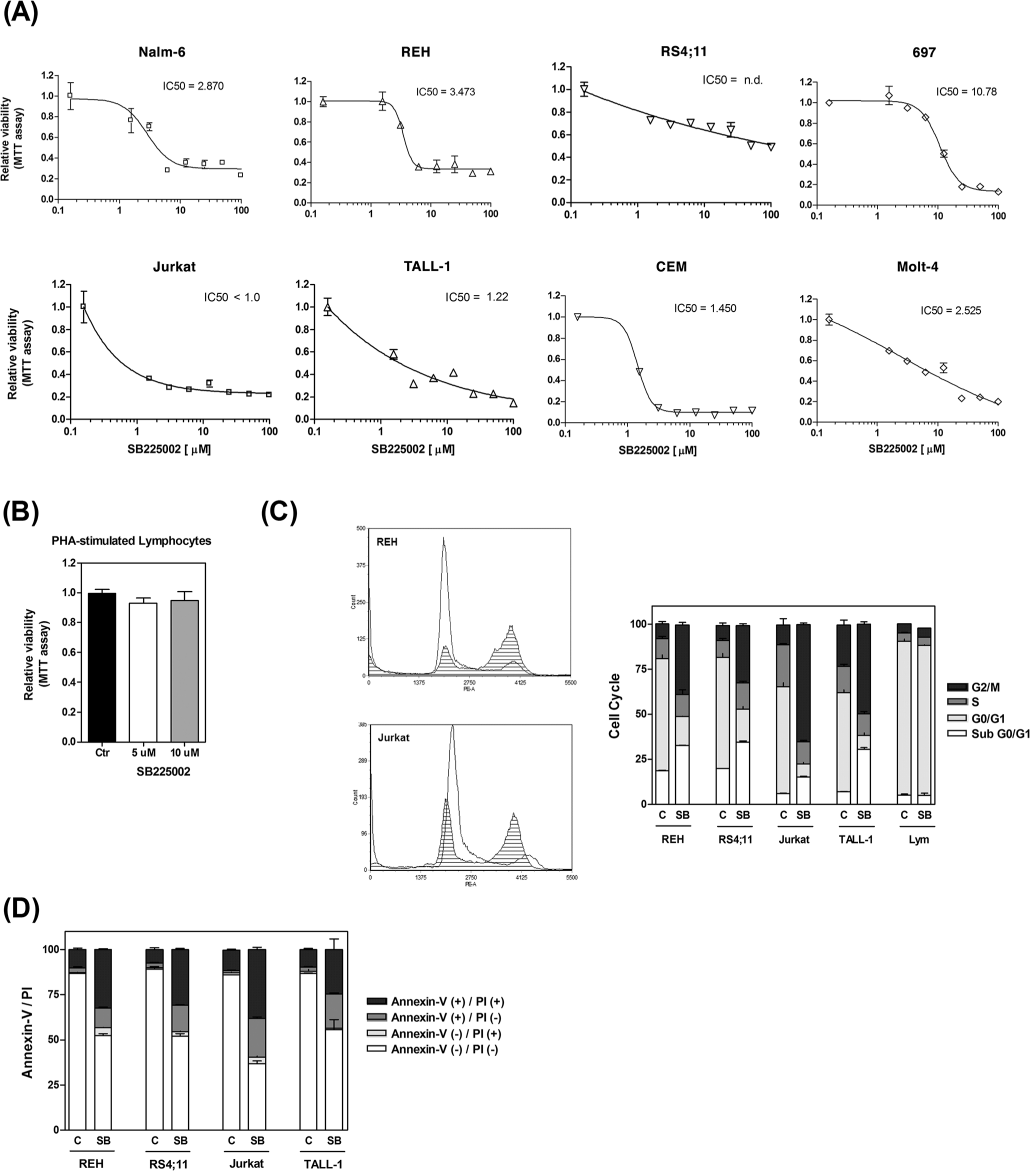
SB225002 induces cell death in ALL cell lines. Effect of SB225002 [100 to 1.5625 μM] on the survival and proliferation of **(A)** B-ALL and T-ALL cell lines. **(B)** Effect of SB225002 [5 and 10 μM] on the survival and proliferation of normal PHA-stimulated human lymphocytes. **(C)** Cell cycle analysis of B-ALL (REH and RS4;11), T-ALL (Jurkat and TALL-1) and normal human PHA-stimulated lymphocytes treated with DMSO (vehicle; 0.1%) and the following concentrations of SB225002: REH and RS4;11 [10 μM]; Jurkat and TALL-1 [3.125 μM]; PHA-stimulated lymphocytes [10 μM]. Representative PI-staining histograms of cells treated with vehicle (clear area) or SB225002 (shaded area) are shown. **(D)** Annexin-V and propidium iodide flow cytometry analyses of B-ALL (REH and RS4;11) and T-ALL (Jurkat and TALL-1) treated with DMSO (vehicle; 0.1%) and SB225002 [10 μM]. Cells were treated for 24 h (for cell cycle and Annexin-V analyses) and 48 h (for MTT analysis). ALL = acute lymphoblastic leukemia; PI = propidium iodide; Lym = PHA-stimulated lymphocytes; C or Ctr = DMSO (vehicle control); SB = SB225002 treatment.

As shown in Fig 1A, micromolar concentrations of SB225002 consistently inhibited the proliferation of the different ALL cell lines tested. In general, T-ALL cell lines (Jurkat, TALL-1, CEM and Molt-4) appeared to be more sensitive to SB225002 than precursor-B ALL cell lines (Nalm-6, REH, RS4;11 and 697). To investigate the potential cytotoxic effects of SB225002 on normal non-cancerous cells, we treated PHA-stimulated normal lymphocytes with SB225002 [5 μM and 10 μM]. As shown in Fig 1B, no cytotoxic effects were observed in the normal lymphocytes at these doses.

Primary ALL cells die rapidly when cultured *in vitro* [27]. Therefore, the effect of SB225002 on primary ALL was evaluated in a xenograft model of B-ALL. Balanced cohorts (n = 4 mice per treatment group) with established disease (% ALL cells ≥ 0.5% in peripheral blood) were treated with vehicle (control group) or SB225002 (10 mg/Kg intraperitoneally once a day, 5 days a week, during 4 weeks). Mice treated with SB225002 as single agent demonstrated a very modest trend towards prolonged overall survival compared to vehicle-treated controls (S1 Fig).

### SB225002 treatment resulted in cell cycle arrest at G2/M and apoptosis of ALL cell lines

Recently, SB225002 was shown to possess a microtubule destabilizing activity, accompained by suppression of microtubule polymerization and induction of a prometaphase arrest [4]. It was also shown to promote mitotic catastrophe in ovarian cancer cells [5]. Cell cycle analysis in ALL cells treated with SB225002 (REH and RS4;11 [10 μM], Jurkat and TALL-1 [3.125 μM]) resulted in G2/M arrest (Fig 1C). In contrast, no cell cycle effects were observed in the PHA-stimulated lymphocytes upon treatment with 10 μM SB225002 (Fig 1C). Analysis of mitotic ALL cells under SB225002 treatment (IC_50_ dose) showed none of the abnormalities suggestive of mitotic catastrophe (spindle abnormalities, chromosome mis-segregation, multi-polar cell division, multiple nuclei, aneuploidy/polyploidy; data not shown).

To determine if the deleterious effects of SB225002 in ALL were caused by the induction of apoptosis, we performed Annexin-V/Propidium Iodide (PI) analysis in B-ALL (REH and RS4;11) and T-ALL (Jurkat and TALL-1) cells treated with SB225002 [10 μM] for 24 h. Most of the untreated ALL cells were non-apoptotic (Annexin-V negative/PI negative), while upon treatment with SB225002 a marked increase in the number of early (Annexin-V positive/PI negative) and late apoptotic (Annexin-V positive/PI positive) cells was observed in both B- and T-ALL cells (Fig 1D).

### The transcriptional profile elicited by SB225002 is similar to that of tubulin inhibitors and involves the *JUN*, *p53* and *TNF* pathways

To get more insights into the mode of action of SB225002, gene expression profiling analysis was performed in Jurkat cells treated with SB225002 [IC_50_ dose] for 6 h and 9 h. Transcriptional profiling analysis revealed 174 induced and 41 repressed genes that were commonly modulated after both 6 h and 9 h of treatment (S1 Table).

**Fig 2:**
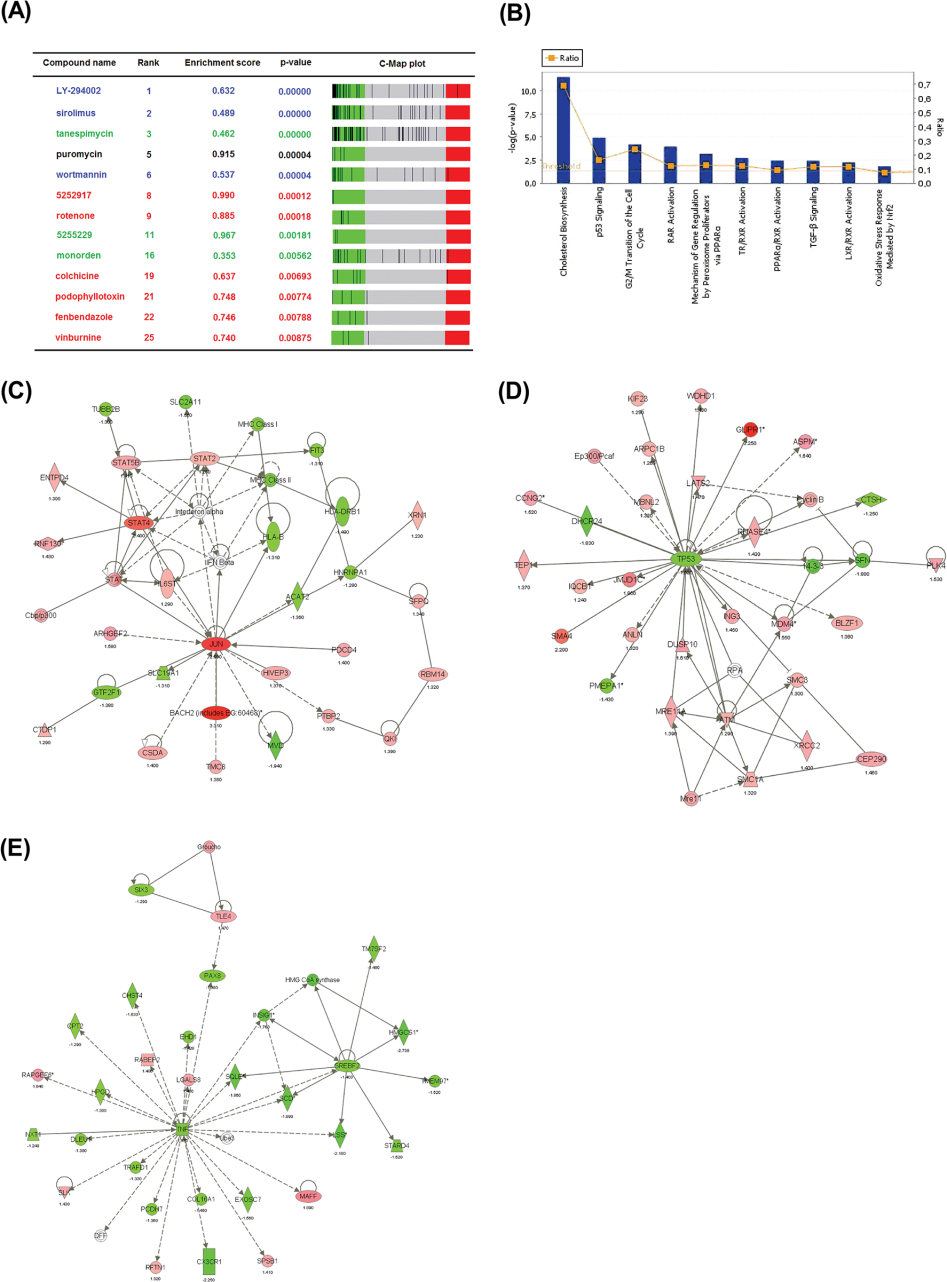
Connectivity Map and Ingenuity Pathway Analysis using the SB225002-derived gene expression signature. **(A)** Connectivity Map (C-Map) analysis using the gene expression signature of Jurkat cells treated with SB225002 [IC_50_] for 9 h. Compounds colored as black bars in each respectively C-Map plot. Compounds are color-coded as follows: blue, PI3K/mTOR inhibitors; green, HSP90 inhibitors; red, tubulin inhibitors. **(B)** Signaling pathways activated in Jurkat cells in response to 6 h of SB225002 [IC_50_] treatment. The statistical threshold (line without boxes) represents the cut-off for significance on the log scale (*y*-axis, left side). The ratio (line with boxes) of the number of significant genes from the data set that mapped to a pathway divided by the total number of genes from the pathway is also shown (*y* axis, right side). **(C)** *JUN*, **(D)** *p53* and **(E)** *TNF* pathways are modulated in Jurkat cells after 6 h of SB225002 [IC_50_] treatment. Analyses were performed using the Ingenuity Pathways Analysis package (Ingenuity Systems).

The gene expression signature of Jurkat cells treated during 9 h with SB225002 was compared to the Connectivity Map (C-Map) database (build 02), which included 6,100 genome-wide expression profiles representing 1,309 compounds. As shown in Fig 2A, the transcriptome effect of SB225002 showed high similarity to inhibitors of the PI3K/mTOR pathway (LY-294002, sirolimus, and wortmannin), inhibitors of the HSP90 chaperone (tanespimycin, 5255229, and monorden) and tubulin binding agents (5252917, rotenone, colchicine, podophyllotoxin, fenbendazole, and vinburnine). Importantly, 6 out of the top 17 compounds positively associated with the SB225002 signature were tubulin inhibitors. Jurkat and REH cells treated with SB225002 showed no alterations in p-PDK1 Ser241, p-AKT Ser473, and p-GSK3beta Ser9 levels (S2 Fig). On the other hand, cell cycle arrest at G2/M (Fig 1C), and suppression of microtubule polymerization [4] are in agreement with SB225002 targeting of tubulin.

The differentially expressed genes after 6 h of treatment (before cell death was observed) were also analyzed using Ingenuity Pathway Analysis. As shown in Fig 2B, SB225002 treatment interfered with the cellular metabolism of cholesterol, p53 signaling, cell cycle progression, nuclear receptor signaling, TGF-β signaling and oxidative stress response. Interactive network analysis of the differentially expressed genes suggested that SB225002 triggered a transcriptional program of genes related to the activation of *JUN* and *p53* pathways and inhibition of *TNF* pathway (Fig 2C–2E).

To validate these findings, we performed quantitative PCR (Q-PCR) analysis on selected candidate genes that appeared to be modulated by SB225002 treatment. The *JUN* pathway is directly and indirectly involved in apoptosis induction [28], and the transcription factor *BACH2* is one of the most important effectors of *JUN* pathway apoptosis response [29]. Q-PCR assays confirmed that both *c-JUN* and *BACH2* transcripts are up-regulated in Jurkat cells after 6 h and 9 h of treatment with SB225002 (S3 Fig). Since Western blot analyses of c-JUN and BACH2 in REH and Jurkat cells rendered inconclusive results (data not shown), this pathway was not further explored in this work.

**Fig 3:**
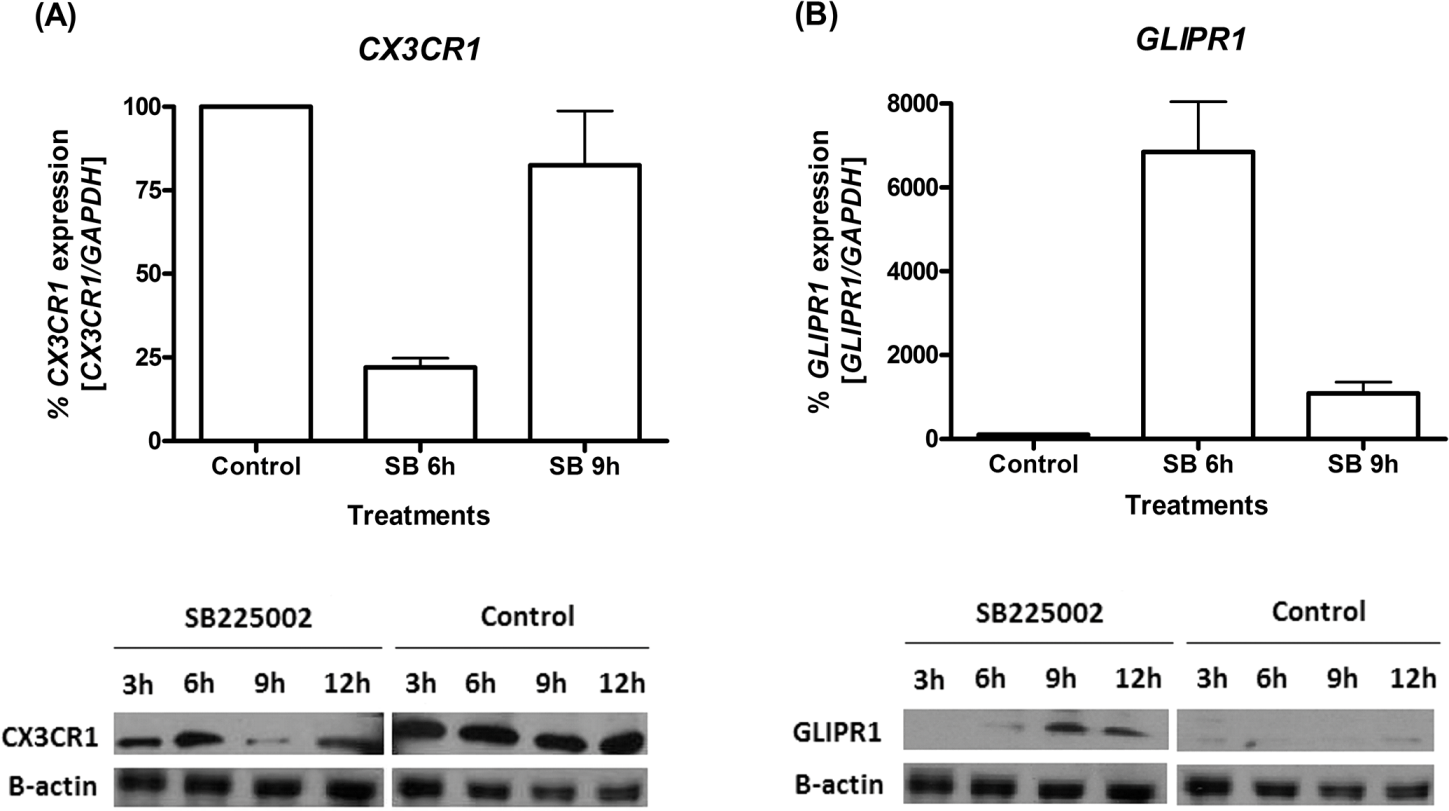
Modulation of *CX3CR1* and *GLIPR1* expression in ALL cells upon SB225002 treatment. **(A)** *CX3CR1* and **(B)** *GLIPR1* gene expression analysis by quantitative PCR (Q-PCR) and Western blot in Jurkat cells treated with SB225002 [IC_50_] or DMSO (vehicle control; 0.1%). Treatments were performed for 3h, 6 h, 9 h or 12 h, as indicated. In the Q-PCR analysis, expression values were calculated considering vehicle control (DMSO) as 100%. β-actin was used as loading control in Western blot analysis. Control = DMSO (vehicle control); SB = SB225002 treatment.

As shown in Fig 2E, SB225002 inhibited several targets of the *TNF* pathway. *CX3CR1*, a *TNF* target found to be down-regulated by SB225002 treatment, has been implicated in chronic lymphocytic leukemia attraction and adhesion to bone marrow stromal cells [30]. Q-PCR and Western blot analyses demonstrated a consistent inhibition of *CX3CR1* transcripts after SB225002 treatment (Fig 3A). Interestingly, while Q-PCR demonstrated *CX3CR1* transcripts more strongly down-regulated at the 6 h timepoint, Western blot analysis showed that protein levels were mostly down-regulated at the 9 h timepoint, but in both cases demonstrating that CX3CR1 was modulated by the SB225002-treatment.

As shown in Fig 2D, *GLIPR1* was among the highest up-regulated genes in the *p53* network modulated by SB225002 treatment. Q-PCR and Western blot analyses confirmed that treatment of Jurkat cells with SB225002 induced activation of GLIPR1 both at the mRNA and protein level (Fig 3B).

### SB225002-mediated cell death is at least in part dependent on the activation of *GLIPR1*

**Fig 4:**
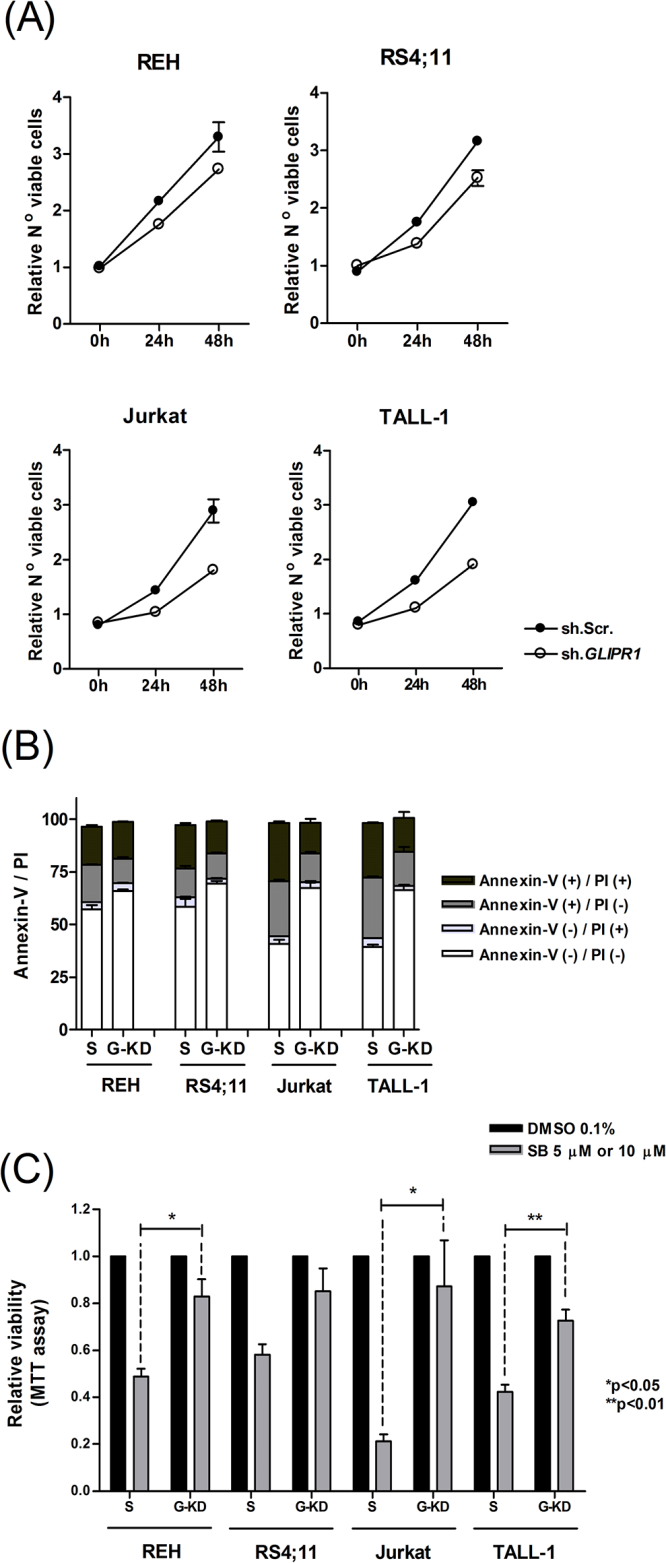
SB225002 induced cell death in ALL is mediated at least in part by the upregulation of *GLIPR1*. **(A)** Relative proliferation of REH, RS4;11, Jurkat and TALL-1 cells upon sh.RNA knockdown of *GLIPR1* (sh.GLIPR1) in comparison to control Scramble (sh.Scr). Number of viable cells were counted by flow cytometry and normalized to time-point zero. **(B)** Annexin-V and propidium iodide flow cytometry analyses of B-ALL (REH and RS4;11) and T-ALL (Jurkat and TALL-1) scramble or *GLIPR1*-knockdown cells treated with SB225002 [5 μM]. **(C)** Effect of SB225002 [5 μM or 10 μM] treatment on the proliferation of *GLIPR1*-knockdown (sh.*GLIPR1*) *versus* control (sh.Scramble) B-ALL (REH and RS4;11) and T-ALL (Jurkat and TALL-1) cell lines. B-ALL cells were treated with SB225002 [10 μM] and T-ALL with SB225002 [5 μM]. Treatment control was DMSO 0.1%. Cells were incubated for 48 h. S = scramble transfection control; G-KD = cells infected with *GLIPR1*-shRNA lentiviral particles (Sigma-Aldrich). P values were calculated using two-tailed Student’s t-test.

*GLIPR1* plays a pro-apoptotic role in prostate and bladder cancer cells [31]. In contrast, in other tumor types such as glioblastoma, *GLIPR1* over-expression is associated with an increase in cellular proliferation and tumor invasion [32]. These data suggest that GLIPR1 effects in cancer cells are dependent on the tumor type. To investigate the relevance of *GLIPR1* up-regulation on SB225002-mediated cell death in ALL, B-ALL (REH and RS4;11) and T-ALL (Jurkat and TALL-1) cells were transduced with *GLIPR1*-shRNA or control-shRNA (scramble) lentiviral particles. Two *GLIPR1*-shRNA clones demonstrated equivalent results in the effectiveness of down-regulating *GLIPR1* when screened in Jurkat cells (S4A Fig). Clone #2 (TRCN0000123176, Sigma-Aldrich) was chosen for the subsequent experiments. Knockdown of *GLIPR1 (GLIPR1*-KD) in four different ALL cell lines was confirmed by Q-PCR (S4B Fig). *GLIPR1*-KD resulted in reduced proliferation in all the ALL cell lines as analyzed by two independent methods (Fig 4A; number of viable cells measured by flow cytometry and S5A Fig; number of viable cells measured by MTT). *GLIPR1*-KD alone did not induce apoptosis in ALL cells lines compared to the scramble control cells in different timepoints (S5B Fig). When both *GLIPR1*-KD and scramble control cells were treated with SB225002 [1.25 μM or 5 μM] for 24 h, a slighty higher number of non-apoptotic (Annexin-V negative/PI negative) and a smaller number of early (Annexin-V positive/PI negative) and late apoptotic (Annexin-V positive/PI positive) cells were observed in *GLIPR1*-KD cells compared to scramble controls. This difference was more markedly observed in the T-ALL cells (Fig 4B and S6 Fig). In addition, *GLIPR1*-KD resulted in a significant attenuation of the inhibitory effect of SB225002 on cell proliferation (Fig 4C).

**Fig 5:**
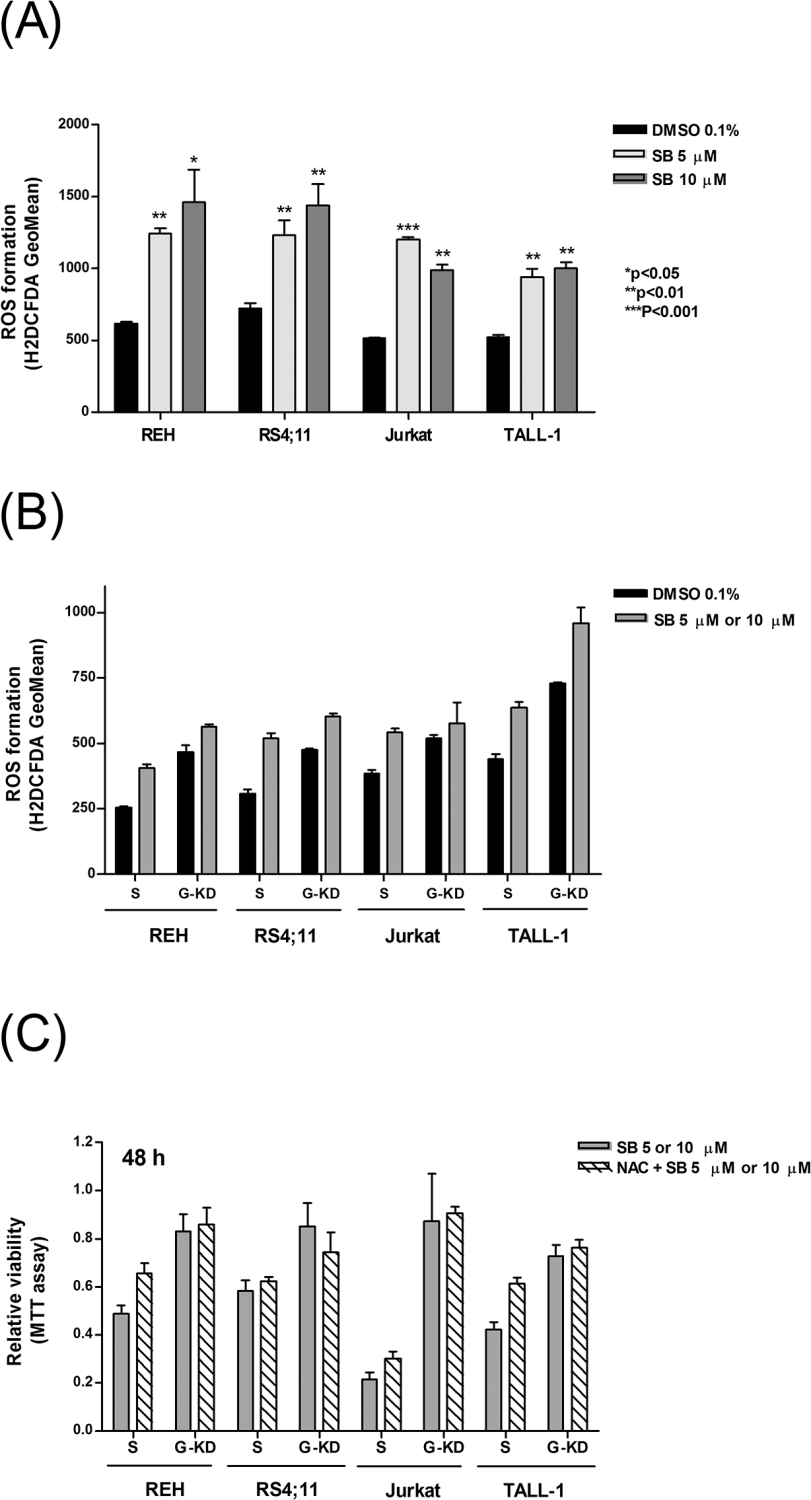
SB225002 and *GLIPR1* knockdown effects on ROS generation in ALL cells. **(A)** Reactive oxygen species production in B-ALL (REH and RS4;11) and T-ALL (Jurkat and TALL-1) cells treated with DMSO (vehicle; 0.1%) and SB225002 [5 μM and 10 μM]. Cells were treated for 24 h. **(B)** ROS generation in *GLIPR1*-knockdown (G-KD) *versus* control scramble (S) cells treated with DMSO (vehicle; 0.1%) or SB225002 [10 μM] for REH and RS4;11 or SB225002 [5 μM] for Jurkat and TALL-1 for 24 h. **(C)** Effect of N-Acetyl Cysteine (NAC; a ROS scavenger) pre-treatment on the survival of *GLIPR1*-knockdown (G-KD) *versus* control scramble (S) ALL cell lines upon SB225002 treatment for 48 h. Cells were pre-incubated or not with NAC [10 mM] for 3h prior to the SB225002 treatment. SB225002 was used at [10 μM] (REH and RS4;11) or [5 μM] (Jurkat and TALL-1). S = scramble transfection control; G-KD = cells infected with *GLIPR1*-shRNA lentiviral particles (Sigma-Aldrich). P values were calculated using two-tailed Student’s t-test.

Apoptosis induction mediated by *GLIPR1* is reported to be at least in part dependent on the production of reactive oxygen species (ROS) [33]. To investigate the potential increase on ROS generation upon SB225002 treatment, B-ALL (REH and RS4;11) and T-ALL (Jurkat and TALL-1) cells treated with SB225002 [5 μM and 10 μM] for 24 h were assessed for ROS formation by H_2_DCFDA labeling followed by flow cytometry analysis. As shown in Fig 5A, SB225002 treatment induced ROS production in ALL cells. For comparison, ROS formation was assessed in PHA-stimulated normal lymphocytes treated with SB225002 [5 μM and 10 μM]. No effect in the production of ROS was observed (S7 Fig).

To determine whether ROS production was promoted, at least in part, by *GLIPR1* expression, *GLIPR1*-KD cells were analyzed concerned the production of ROS. Surprisingly, ROS production was higher in the *GLIPR1*-KD than in the scramble control cell lines, and SB225002 treatment resulted in increased ROS generation, even in *GLIPR1*-KD cells (Fig 5B). Incubation of ALL cells in presence of a ROS scavenger, N-Acetyl Cysteine (NAC), resulted in short term (6 h) decrease in ROS generation (S8 Fig) but no attenuation of SB225002 effects in terms of cell viability (Fig 5C). All together, these data suggest that the pro-apoptotic function of *GLIPR1* in ALL cells, upon SB225002 treatment, is not linked to ROS generation.

## Discussion

This study shows for the first time an anti-proliferative and pro-apoptotic activity of SB225002 against acute lymphoblastic leukemia. SB225002 treatment of leukemia cells induced ROS generation and cell cycle arrest at G2/M. Microarray expression analysis of SB225002-treated Jurkat cells revealed a transcriptional program typically triggered by tubulin binding agents, with some degree of overlap to the gene expression signature derived from PI3K/mTOR and HSP90 inhibitors. Suppression of microtubule polymerization by SB225002 has been experimentally demonstrated [4] and was therefore favored in our interpretation of possible molecular mechanisms for the connection between SB225002 and PI3K/mTOR or HSP90 inhibitors. For instance, HSP90 protects tubulin, keeping it in a state compatible with microtubule polymerization [34], thus the transcriptional program elicited by HSP90 inhibitors would be expected to overlap, at least in part, the one elicited by tubulin inhibitors.

Two drugs may elicit similar transcriptional profiles acting on different cellular targets. We speculate that the connection between SB225002 and PI3K/mTOR or HSP90 inhibitors may be related to the effects of anti-microtubule agents on the endoplasmic reticulum (ER) and/or translation machinery. Consistent with this hypothesis, Puromycin, an inhibitor of protein translation was also strongly linked to SB225002 in the C-Map analysis (Fig 2A).

Since ribosomal proteins, translation initiation factors, and other components of the translation machinery are associated with the cytoskeleton [35, 36], destabilization of microtubules have a drastic effect on protein synthesis. Both inhibition of the PI3K/mTOR pathway [37] and inhibition of microtubule polymerization [36] result in the phosphorylation and inactivation of the translation initiation factor eIF2α. Inactivation of eIF2α causes a generalized translation repression, except for some few transcripts, including the ATF4 transcription factor, that are able to recruit ribosome binding to internal ribosome entry sites (IRES). Likewise, tubulin inhibition and disruption of the actin cytoskeleton by Cucurbitacin E were shown to increase eIF2α phosphorylation, inhibiting protein synthesis [38].

Microtubule dynamics is important to ER homeostasis. Diverse stressful conditions, including ER stress, converge to translation attenuation via eIF2α phosphorylation [39]. Microtubule disruption by colchicine leads to ER collapse and deposition of large perinuclear protein aggregates [40]. Taxol and vinblastine were shown to induce the ER stress response, including eIF2α phosphorylation [41]. Misfolded proteins resulting from HSP90 inhibitors also trigger the ER stress response [42]. Likewise, interruption of mRNA translation by Puromycin leads to the accumulation of truncated, misfolded proteins [43], and ER stress [44].

SB225002 treatment of ALL cells resulted in remarkable downregulation of cholesterol biosynthesis genes (Fig 2B and S1 Table). This transcriptional output of SB225002 could be also attributed to ER stress. In fact, ER stress has been reported to cause significant transcriptional down-regulation of genes encoding key enzymes in cholesterol biosynthesis [45]. Modulation of cholesterol biosynthesis genes may also underlie the connection between SB225002 and PI3K/mTOR inhibitors. We have recently found that the top biological functions downregulated by the PI3K inhibitor AS605240 in T-ALL (cell lines and primary cells) are related to cholesterol biosynthesis [46]. Likewise, the mTOR inhibitor Everolimus downregulated several lipid and fatty acid biosynthesis genes and induced ER stress genes in different ALL cell lines [47].

Transcriptome network analysis revealed that SB225002-induced cell death was associated to the activation of *JUN* and *p53* pathways, and inhibition of the *TNF* pathway. Although the human T-ALL Jurkat cell line contains a non-sense mutation in its *p53* gene, it still preserves a functional p53 protein [48, 49]. *GLIPR1*, a transcriptional target of p53 [31, 50] that was significantly upregulated upon SB225002 treatment, was chosen for further investigation.

*GLIPR1* has been associated with variable functions in humans, such as cell proliferation, apoptosis and tumor growth [51]. In prostate cancer, increased expression of *GLIPR1* is associated with apoptosis induction [31]. In contrast, in astrocytes-derived tumors over-expression of *GLIPR1* is associated with an enhancement in cellular proliferation and tumor invasion, while *GLIPR1* silencing is associated with an elevated level of apoptosis [32]. Recently, activation of *GLIPR1* with an adenoviral clinical vector, in association with radiotherapy, significantly suppressed tumor growth and extended survival in prostate and bladder cancer *in vivo* models, suggesting that *GLIPR1* activation should be explored as a potential therapeutic strategy at least in prostate and bladder tumors [52]. *GLIPR1* is significantly underexpressed in ALL when compared to normal controls [53], which is compatible with a pro-apoptotic role of *GLIPR1* activation upon SB225002 treatment. This is the first time functional assays were performed to evaluate the role of *GLIPR1* in ALL. Importantly, down-regulation of *GLIPR1* in B- and T-ALL cells resulted in decreased proliferation of ALL cells and in a significant increase in cellular resistance to SB225002 treatment. Since SB225002 has an anti-mitotic effect, increased resistance to SB225002 upon *GLIPR1* silencing could simply reflect the lower rate of cell proliferation. However, cytoskeleton disruption by tubulin binding agents do not spare interphase cells [54]. In addition, it has been shown that in prostate cancer cells there is an inverse correlation between the expression of *GLIPR1* and *c-Myc*, where restoration of *GLIPR1* expression downregulates *c-Myc* and induces cell-cycle arrest [55]. It is possible that *GLIPR1* knockdown in ALL cells elevates *c-Myc* levels, leading to an increased resistance to SB225002’s treatment. As discussed above, SB225002-treated cells revealed a gene expression profile suggestive of significant ongoing stress. Therefore, it is not possible to exclude that the cellular effects of SB225002 are at least partially dependent on *GLIPR1* activation. For instance, *GLIPR1* overexpression has been shown to cause inactivation of Bcl-2, dysfunction of mitochondria, and activation of a wide spectrum of caspases [33].

Apoptosis induction upon *GLIPR1* overexpression in a tet-on stable clone of bladder carcinoma cell line was reported to be dependent on the production of ROS [33]. Here, we detected a significant increase in ROS levels in ALL cells after treatment with SB225002. However, upon *GLIPR1* knockdown and treatment with SB225002, none of the cell lines showed a reduction in the production of ROS. In addition, pre-incubation of the cells with N-Acetyl Cysteine, a known ROS scavenger, prior to the treatment with SB225002, did not attenuate cell death. These data suggest that *GLIPR1* gene plays a role in the apoptosis induction mediated by SB225002, but likely not through the modulation of ROS levels in ALL cells. Overall, we believe that SB225002 might have the potential to exert its activity through distinct mechanisms depending on the cellular type. Further studies including systems biology approaches should be considered and will be useful for better understanding of SB225002’s effects and downstream molecular events in ALL.

Finally, the treatment of a xenograft model of ALL with SB225002 demonstrated a trend towards prolonged overall survival compared to vehicle-treated controls. These are preliminary, but encouraging results, which suggest further studies with SB225002 *in vivo*, with larger animal cohorts, increased treatment dose(s) and leukemia cells from different patients to better represent the heterogeneity of the disease, should be considered.

In conclusion, our results demonstrate that SB225002 has anti-proliferative and pro-apoptotic effects against precursor B- and T-ALL cell lines, at the micromolar concentration range. Cells treated with SB225002 undergo cell cycle arrest at G2/M and exhibited a transcriptional gene expression profile typically elicited by tubulin binding agents. SB225002-mediated cell death is at least partially dependent upon *GLIPR1* up-regulation, irrespective of ROS generation.

## Supporting Information

S1 Fig Effects of SB225002 in the progression of ALL *in vivo*. **(A)** Kinetics of human hCD45(+) cells in the peripheral blood of mice transplanted with primary xenograft ALL and treated with vehicle or SB225002 [10 mg/Kg] intraperitoneally, once a day, 5 days a week, during 4 weeks. **(B)** Kaplan-Meier survival curve of mice treated with vehicle or SB225002 [10 mg/Kg] as described above. P value was calculated using Log-rank test.
(TIF)

S2 Fig Modulation of p-PDK1 (Ser241), p-AKT (Ser473), and p-GSK3beta (Ser9) levels in ALL cells upon SB225002 treatment. **(A)** REH and **(B)** Jurkat cells protein levels were investigated by Western blot analysis. Cells treated with SB225002 [IC_50_] or DMSO (vehicle control; 0.1%) for 3h, 6 h, 9 h or 12 h, as indicated. GAPDH was used as loading control. Control = DMSO (vehicle control); SB = SB225002 treatment.
(TIF)

S3 Fig Validation of the transcriptional activation of *c-JUN* and *BACH2* in ALL cells upon SB225002 treatment. Gene expression analysis for **(A)** *c-JUN* and **(B)** *BACH2* were performed by quantitative PCR in Jurkat cells. Expression values were calculated considering vehicle control (DMSO) as 100%. Control = DMSO (vehicle control); SB = SB225002 treatment.
(TIF)

S4 Fig Gene expression level by quantitative PCR in Jurkat cells transduced with two different *GLIPR1* sh.RNA clones. Expression values were calculated considering transduction control as 100%. G-KD#1 corresponds to clone TRCN0000123175 and G-KD#2 to clone TRCN0000123176 (both from Sigma-Aldrich). **(B)** Validation of *GLIPR1* knockdown in the different B-ALL (REH and RS4;11) and T-ALL (Jurkat and TALL-1) cell lines performed with clone TRCN0000123176 (Sigma-Aldrich).
(TIF)

S5 Fig Effects of *GLIPR1*-KD on the relative number of viable cells and apoptosis induction of B- and T-ALL cells. **(A)** Relative proliferation of REH, RS4;11, Jurkat and TALL-1 cells upon sh.RNA knockdown of *GLIPR1* (sh.*GLIPR1*) in comparison to control Scramble (sh.Scr). Number of viable cells was measured by the MTT assay and normalized to time-point zero. **(B)** Annexin-V and propidium iodide flow cytometry analyses of B-ALL (REH and RS4;11) and T-ALL (Jurkat and TALL-1) scramble or *GLIPR1*-knockdown cells at different timepoints as indicated. S = scramble transfection control; G-KD = cells infected with *GLIPR1*-shRNA lentiviral particles (Sigma-Aldrich).
(TIF)

S6 Fig Effects of *GLIPR1*-KD on the percentage of viable cells of (A) B-ALL (REH and RS4;11) and (B) T-ALL (Jurkat and TALL-1) treated with SB225002 [1.25 or 5 μM] or DMSO (vehicle control; 0.1%). Cells were treated for 24 h. Percentage of viable cells was analyzed by flow cytometry (annexin-V staining negative population). S = scramble transfection control; G-KD = cells infected with *GLIPR1*-shRNA lentiviral particles (Sigma-Aldrich). P values were calculated using two-tailed Student’s t-test.
(TIF)

S7 Fig Effect on the production of reactive oxygen species in normal human PHA-stimulated lymphocytes treated with DMSO (vehicle; 0.1%) or SB225002 [5 μM and 10 μM]. Cells were treated for 24 h. Control = DMSO (vehicle control); SB = SB225002 treatment.
(TIF)

S8 Fig Effect of N-Acetyl Cysteine (NAC; a ROS scavenger) on ROS generation by *GLIPR1*-knockdown (sh.GLIPR1) *versus* control (sh.Scramble) ALL cell lines upon SB225002 treatment. Cells were pre-incubated with NAC [10 mM] for 3 h prior to the SB225002 treatment. B-ALL (REH and RS4;11) cells were treated with SB225002 [10 μM] and T-ALL (Jurkat and TALL-1) were treated with SB225002 [5 μM] for 6 h or 24 h as indicated. S = scramble transfection control; G-KD = cells infected with *GLIPR1*-shRNA lentiviral particles (Sigma-Aldrich). P values were calculated using two-tailed Student’s t-test.
(TIF)

S1 Table List of induced and repressed genes modulated in both 6 h and 9 h after SB225002 [12.5 μM] treatment. Transcriptional profiling analysis was performed in Jurkat (T-ALL) cells.
(DOCX)
