# Supplementary figures and images for: SB225002 Induces Cell Death and Cell Cycle Arrest in Acute Lymphoblastic Leukemia Cells through the Activation of GLIPR1
Source: PLoS One. 2015 Aug 24;10(8):e0134783. doi: 10.1371/journal.pone.0134783 (PMC4547718; doi:10.1371/journal.pone.0134783)

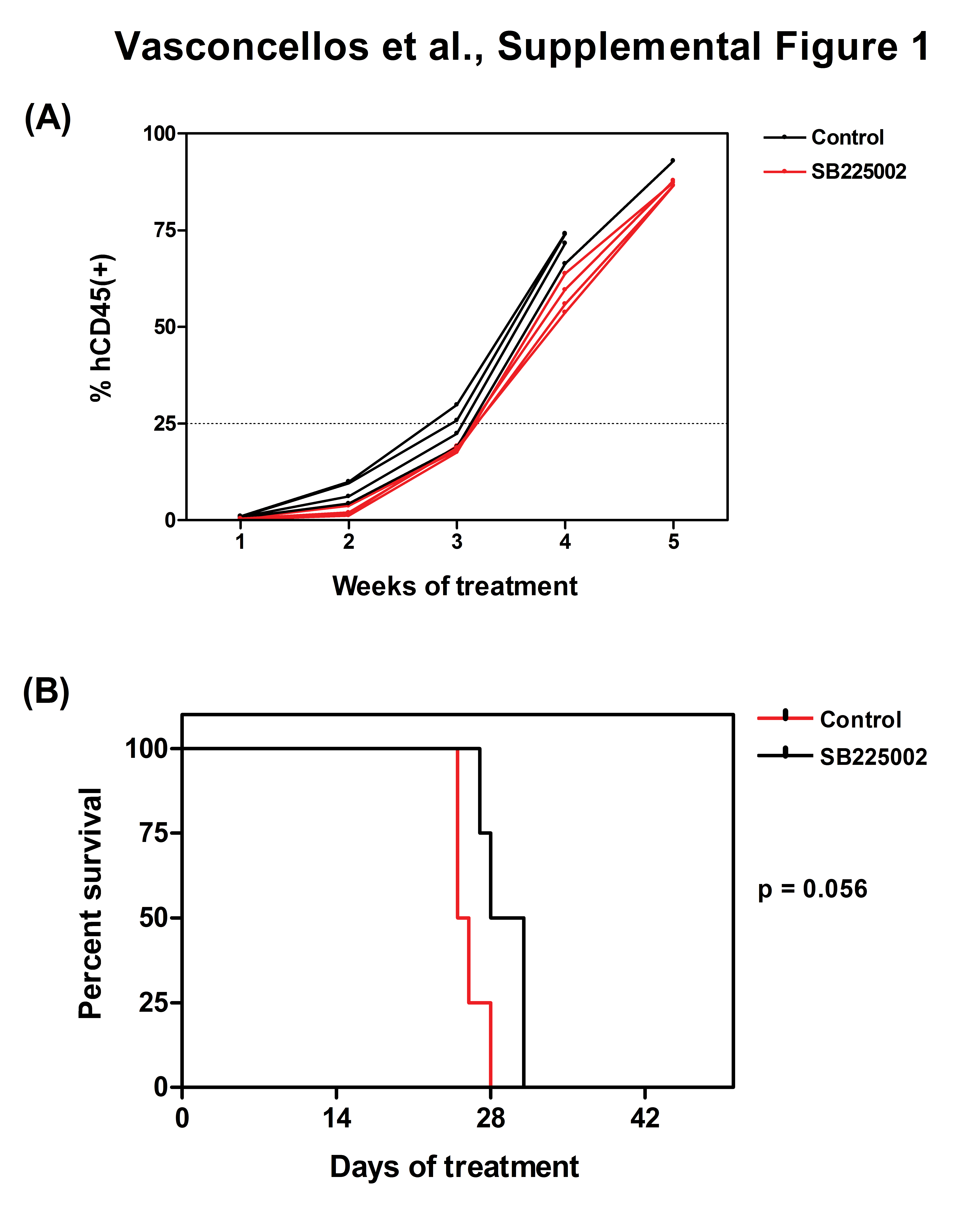

Supplement: S1 Fig — (A) Kinetics of human hCD45(+) cells in the peripheral blood of mice transplanted with primary xenograft ALL and treated with vehicle or SB225002 [10 mg/Kg] intraperitoneally, once a day, 5 days a week, during 4 weeks. (B) Kaplan-Meier survival curve of mice treated with vehicle or SB225002 [10 mg/Kg] as described above. P value was calculated using Log-rank test. (TIF) [file pone.0134783.s001.tif]

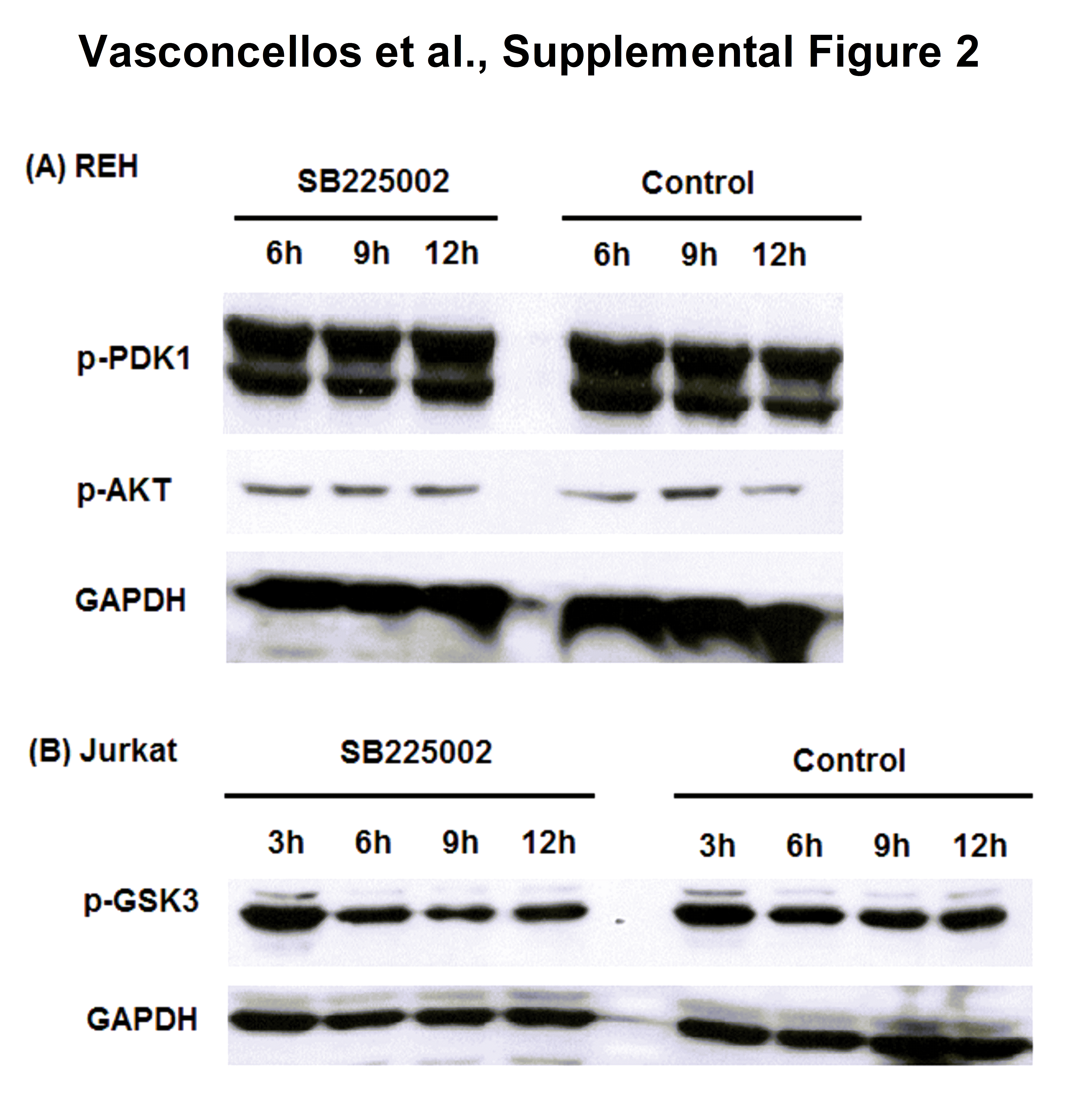

Supplement: S2 Fig — (A) REH and (B) Jurkat cells protein levels were investigated by Western blot analysis. Cells treated with SB225002 [IC50] or DMSO (vehicle control; 0.1%) for 3h, 6 h, 9 h or 12 h, as indicated. GAPDH was used as loading control. Control = DMSO (vehicle control); SB = SB225002 treatment. (TIF) [file pone.0134783.s002.tif]

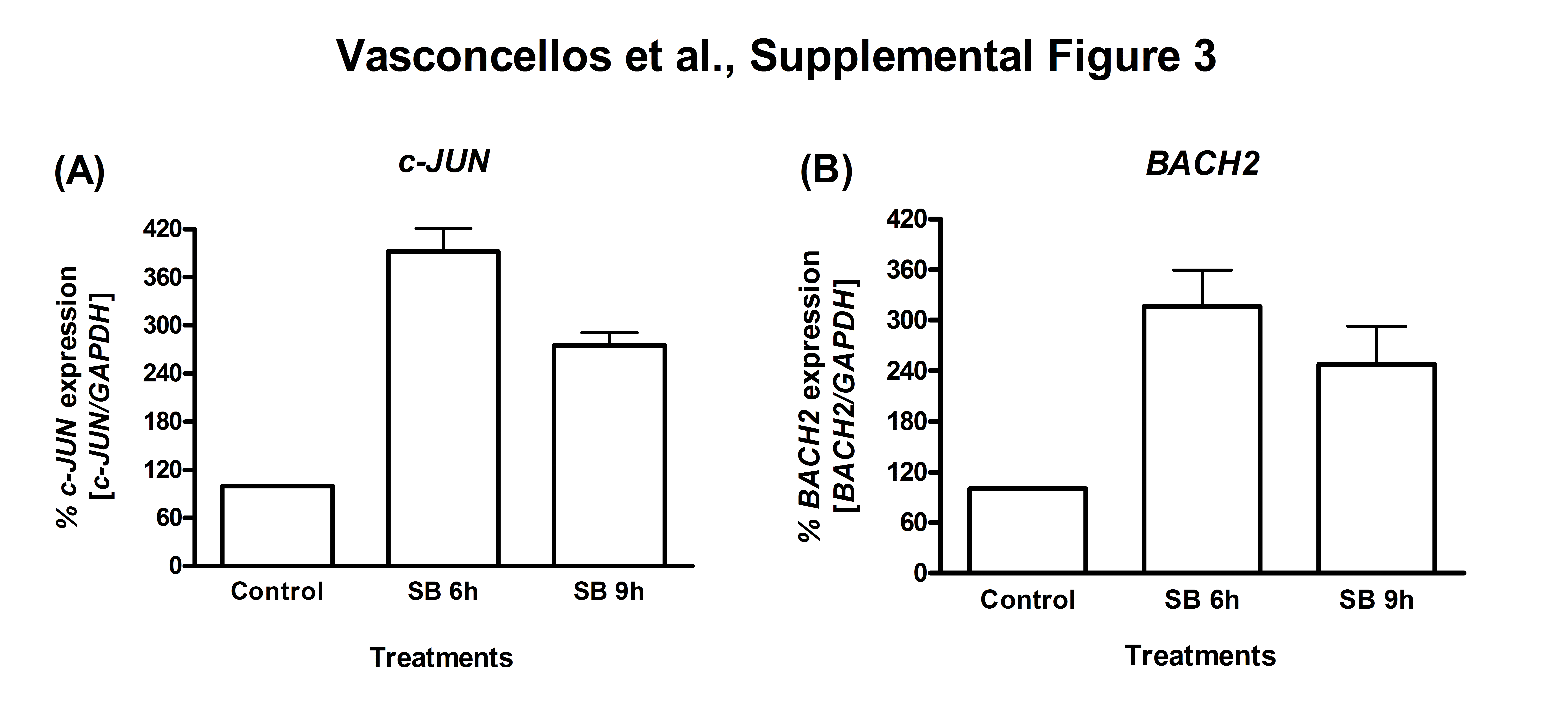

Supplement: S3 Fig — Gene expression analysis for (A) c-JUN and (B) BACH2 were performed by quantitative PCR in Jurkat cells. Expression values were calculated considering vehicle control (DMSO) as 100%. Control = DMSO (vehicle control); SB = SB225002 treatment. (TIF) [file pone.0134783.s003.tif]

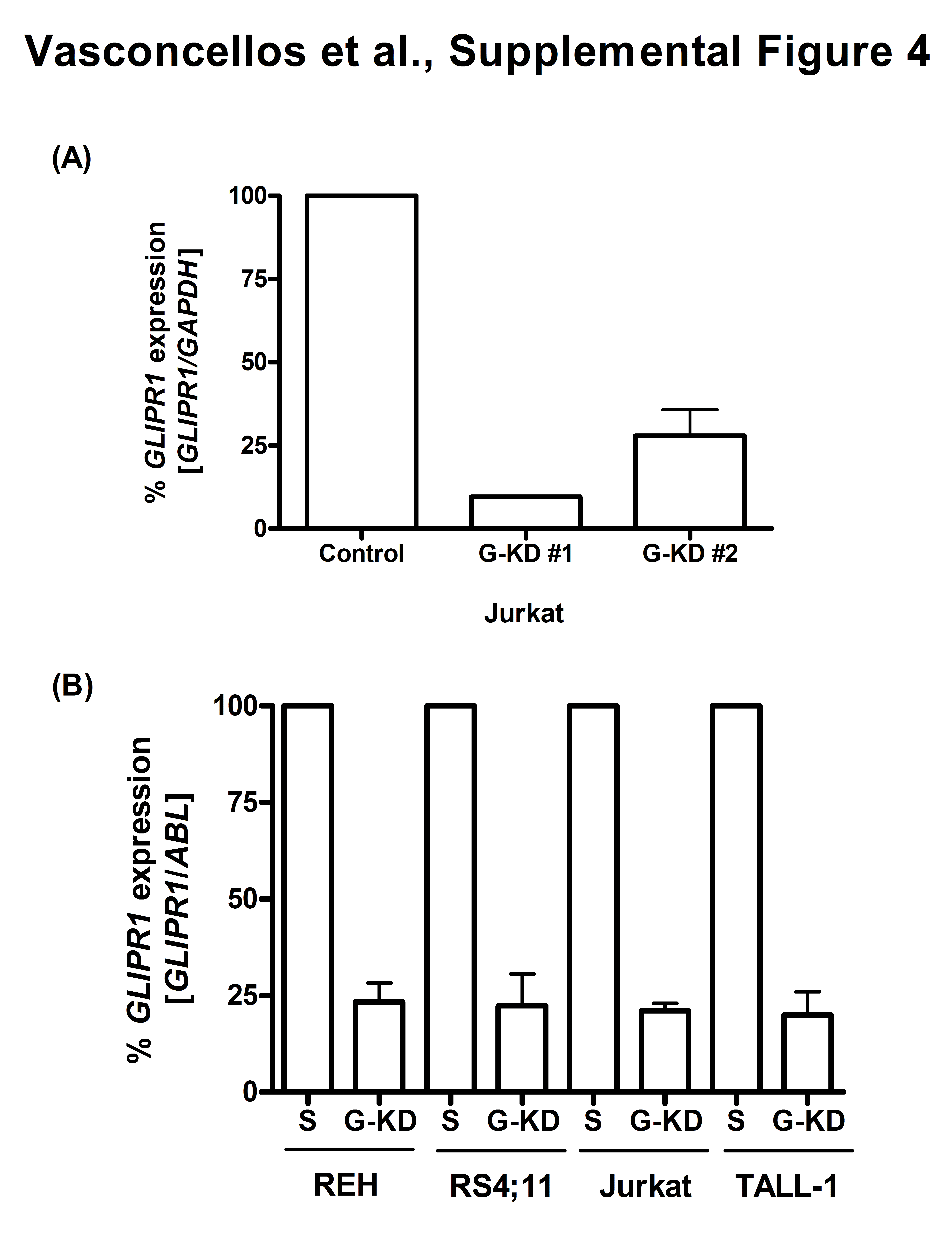

Supplement: S4 Fig — Expression values were calculated considering transduction control as 100%. G-KD#1 corresponds to clone TRCN0000123175 and G-KD#2 to clone TRCN0000123176 (both from Sigma-Aldrich). (B) Validation of GLIPR1 knockdown in the different B-ALL (REH and RS4;11) and T-ALL (Jurkat and TALL-1) cell lines performed with clone TRCN0000123176 (Sigma-Aldrich). (TIF) [file pone.0134783.s004.tif]

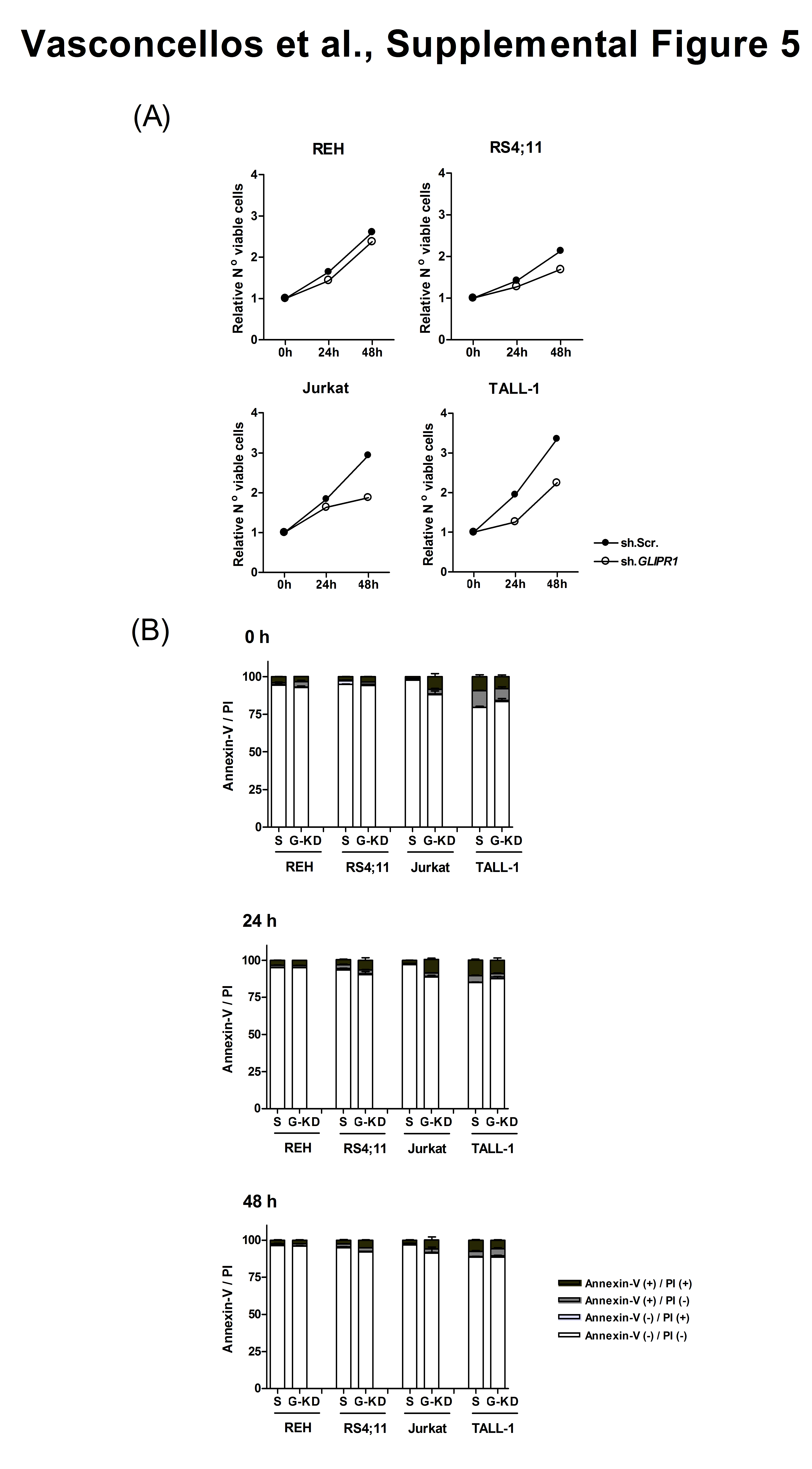

Supplement: S5 Fig — (A) Relative proliferation of REH, RS4;11, Jurkat and TALL-1 cells upon sh.RNA knockdown of GLIPR1 (sh.GLIPR1) in comparison to control Scramble (sh.Scr). Number of viable cells was measured by the MTT assay and normalized to time-point zero. (B) Annexin-V and propidium iodide flow cytometry analyses of B-ALL (REH and RS4;11) and T-ALL (Jurkat and TALL-1) scramble or GLIPR1-knockdown cells at different timepoints as indicated. S = scramble transfection control; G-KD = cells infected with GLIPR1-shRNA lentiviral particles (Sigma-Aldrich). (TIF) [file pone.0134783.s005.tif]

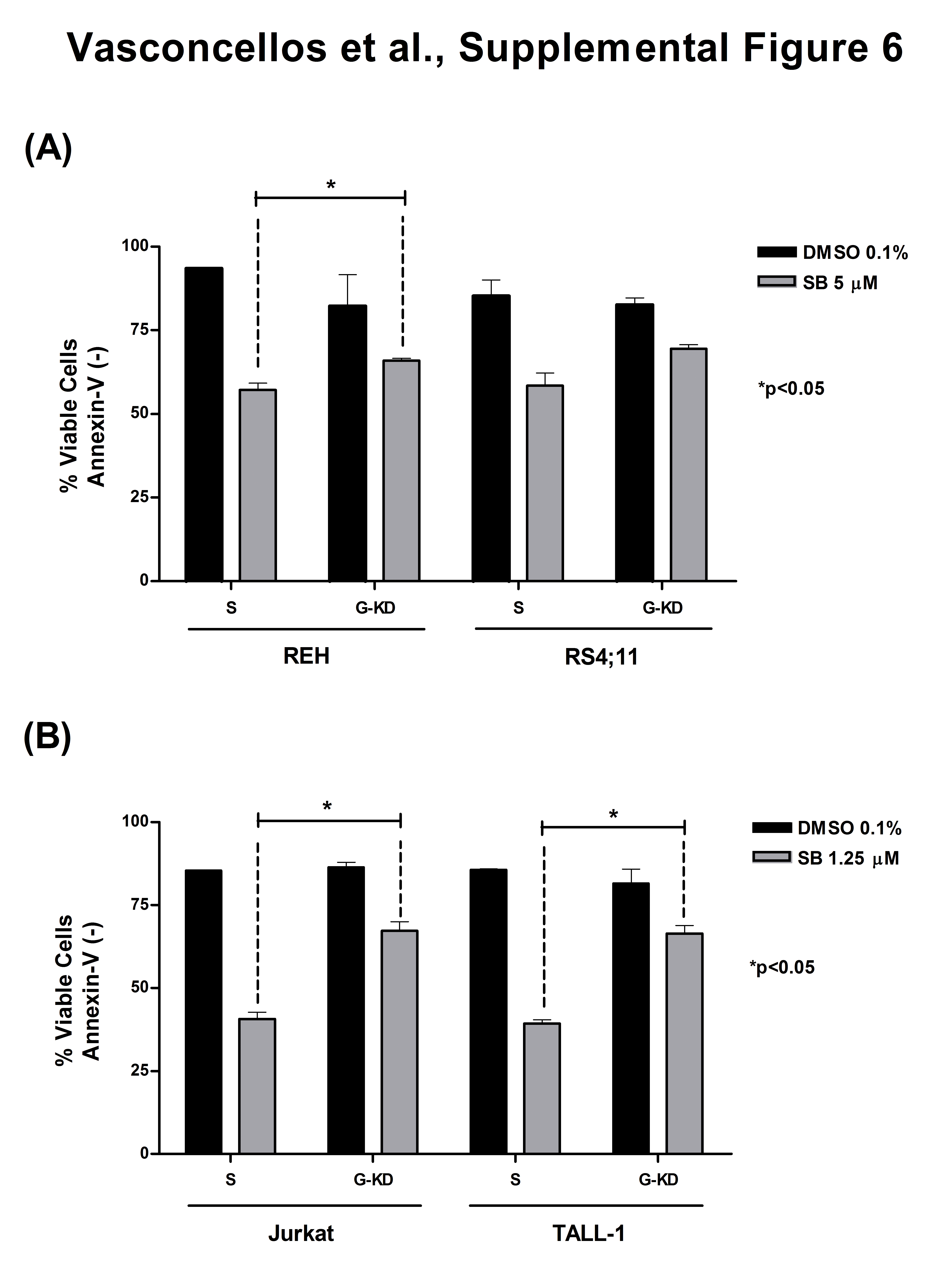

Supplement: S6 Fig — Cells were treated for 24 h. Percentage of viable cells was analyzed by flow cytometry (annexin-V staining negative population). S = scramble transfection control; G-KD = cells infected with GLIPR1-shRNA lentiviral particles (Sigma-Aldrich). P values were calculated using two-tailed Student’s t-test. (TIF) [file pone.0134783.s006.tif]

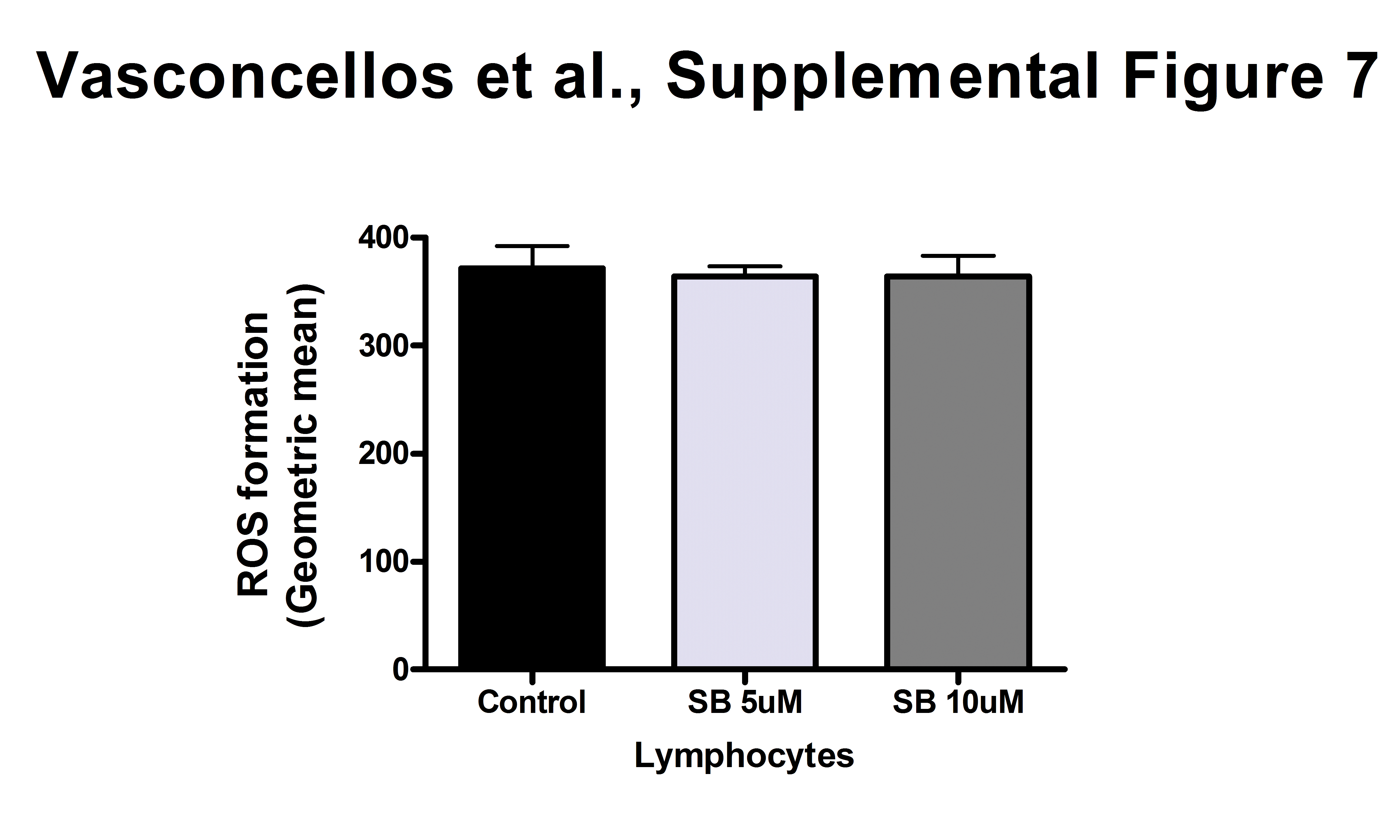

Supplement: S7 Fig — Cells were treated for 24 h. Control = DMSO (vehicle control); SB = SB225002 treatment. (TIF) [file pone.0134783.s007.tif]

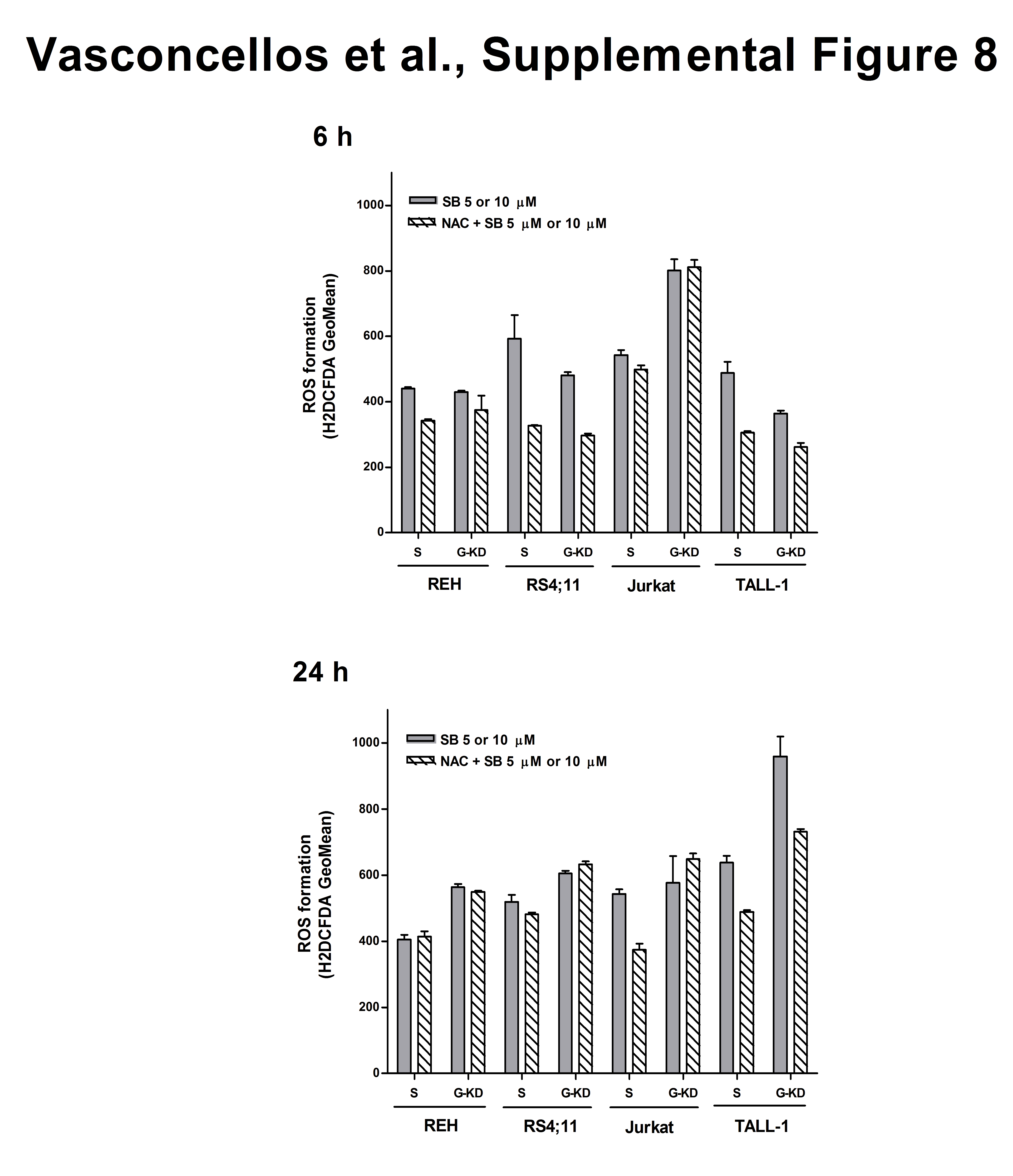

Supplement: S8 Fig — Cells were pre-incubated with NAC [10 mM] for 3 h prior to the SB225002 treatment. B-ALL (REH and RS4;11) cells were treated with SB225002 [10 μM] and T-ALL (Jurkat and TALL-1) were treated with SB225002 [5 μM] for 6 h or 24 h as indicated. S = scramble transfection control; G-KD = cells infected with GLIPR1-shRNA lentiviral particles (Sigma-Aldrich). P values were calculated using two-tailed Student’s t-test. (TIF) [file pone.0134783.s008.tif]
