# Supplementary material for: SB225002 Induces Cell Death and Cell Cycle Arrest in Acute Lymphoblastic Leukemia Cells through the Activation of GLIPR1
Source: PLoS One. 2015 Aug 24;10(8):e0134783. doi: 10.1371/journal.pone.0134783 (PMC4547718; doi:10.1371/journal.pone.0134783)
Supplement: S1 Table — Transcriptional profiling analysis was performed in Jurkat (T-ALL) cells. (DOCX) [file pone.0134783.s009.docx]

**S1 Table-** List of induced and repressed genes modulated in both 6 h and 9 h after SB225002 [12.5 μM] treatment. Transcriptional profiling analysis was performed in Jurkat (T-ALL) cells.

| **Probeset ID** | **Gene symbol** | **Gene name** | **Fold change** |
| --- | --- | --- | --- |
| 244766_at | *LOC440345* | hypothetical protein LOC440345 | 5.36 |
| 238735_at | *---* | *---* | 3.70 |
| 233595_at | *USP34* | ubiquitin specific peptidase 34 | 3.23 |
| 216109_at | *MED13L* | Mediator complex subunit 13-like | 3.27 |
| 226101_at | *PRKCE* | protein kinase C, epsilon | 3.26 |
| 230387_at | *---* | *---* | 3.09 |
| 232889_at | *---* | *---* | 2.82 |
| 233303_at | *---* | *---* | 3.01 |
| 235959_at | *---* | *---* | 2.97 |
| 203910_at | *ARHGAP29* | Rho GTPase activating protein 29 | 2.47 |
| 240452_at | *GSPT1* | G1 to S phase transition 1 | 2.60 |
| 235023_at | *VPS13C* | Vacuolar protein sorting 13 homolog C (S. cerevisiae) | 2.62 |
| 239228_at | *---* | *---* | 2.82 |
| 228105_at | *---* | *---* | 2.44 |
| 242673_at | *---* | *---* | 2.91 |
| 239930_at | *GALNT2* | UDP-N-acetyl-alpha-D-galactosamine:polypeptide N-acetylgalactosaminyltransferase 2 (GalNAc-T2) | 2.32 |
| 242431_at | *---* | *---* | 2.41 |
| 237239_at | *---* | *---* | 2.33 |
| 239735_at | *---* | *---* | 2.21 |
| 213605_s_at | *LOC100134401* | hypothetical protein LOC100134401 | 2.48 |
| 227884_at | *TAF15* | TAF15 RNA polymerase II, TATA box binding protein (TBP)-associated factor, 68kDa | 2.23 |
| 239238_at | *---* | *---* | 2.32 |
| 221234_s_at | ***BACH2*** | BTB and CNC homology 1, basic leucine zipper transcription factor 2 | 2.43 |
| 239937_at | *ZNF207* | Zinc finger protein 207 | 2.21 |
| 238429_at | *TMEM71* | transmembrane protein 71 | 2.07 |
| 240383_at | *UBE2D3* | ubiquitin-conjugating enzyme E2D 3 (UBC4/5 homolog, yeast) | 2.26 |
| 223494_at | *MGEA5* | meningioma expressed antigen 5 (hyaluronidase) | 2.00 |
| 240451_at | *---* | *---* | 2.09 |
| 219906_at | *FLJ10213* | hypothetical protein FLJ10213 | 2.03 |
| 221899_at | *N4BP2L2* | NEDD4 binding protein 2-like 2 | 2.13 |
| 236907_at | *---* | *---* | 2.11 |
| 228613_at | *RAB11FIP3* | RAB11 family interacting protein 3 (class II) | 2.00 |
| 215521_at | *PHC3* | polyhomeotic homolog 3 (Drosophila) | 1.94 |
| 242121_at | *RNF12* | Ring finger protein 12 | 2.24 |
| 226840_at | *H2AFY* | H2A histone family, member Y | 2.32 |
| 242974_at | *CD47* | CD47 molecule | 1.93 |
| 225239_at | *---* | *---* | 2.17 |
| 231956_at | *KIAA1618* | KIAA1618 | 2.23 |
| 234989_at | *TncRNA* | trophoblast-derived noncoding RNA | 2.03 |
| 214964_at | *---* | *---* | 1.82 |
| 243869_at | *---* | *---* | 1.99 |
| 202274_at | *ACTG2* | actin, gamma 2, smooth muscle, enteric | 2.24 |
| 229434_at | *---* | *---* | 1.97 |
| 220197_at | *ATP6V0A4* | ATPase, H+ transporting, lysosomal V0 subunit a4 | 1.92 |
| 231005_at | *---* | *---* | 2.03 |
| 215252_at | *---* | *---* | 2.06 |
| 244808_at | *GRAMD1A* | GRAM domain containing 1A | 1.88 |
| 235927_at | *XPO1* | exportin 1 (CRM1 homolog, yeast) | 2.08 |
| 244185_at | *---* | *---* | 2.14 |
| 215123_at | *LOC348162* | hypothetical protein 348162 | 1.92 |
| 238714_at | *---* | *---* | 1.85 |
| 242550_at | *EIF3B* | eukaryotic translation initiation factor 3, subunit B | 1.77 |
| 228793_at | *JMJD1C* | jumonji domain containing 1C | 2.15 |
| 41577_at | *PPP1R16B* | protein phosphatase 1, regulatory (inhibitor) subunit 16B | 1.82 |
| 240105_at | *---* | *---* | 1.74 |
| 236431_at | *SR140* | U2-associated SR140 protein | 2.18 |
| 224558_s_at | *MALAT1* | metastasis associated lung adenocarcinoma transcript 1 (non-protein coding) | 2.44 |
| 239071_at | *RBBP4* | Retinoblastoma binding protein 4 | 2.01 |
| 235094_at | *---* | *---* | 1.88 |
| 201464_x_at | ***JUN*** | jun oncogene | 2.03 |
| 230237_at | *ADCYAP1* | adenylatecyclase activating polypeptide 1 (pituitary) | 1.68 |
| 225227_at | *---* | *---* | 1.67 |
| 242059_at | *---* | *---* | 1.75 |
| 230761_at | *---* | *---* | 2.09 |
| 214805_at | *EIF4A1* | Eukaryotic translation initiation factor 4A, isoform 1 | 1.84 |
| 223679_at | *CTNNB1* | catenin (cadherin-associated protein), beta 1, 88kDa | 1.91 |
| 242712_x_at | *RANBP2* | RAN binding protein 2 | 1.67 |
| 237246_at | *---* | *---* | 2.26 |
| 235757_at | *---* | *---* | 1.86 |
| 224559_at | *MALAT1* | metastasis associated lung adenocarcinoma transcript 1 (non-protein coding) | 2.30 |
| 215190_at | *EIF3M* | eukaryotic translation initiation factor 3, subunit M | 1.79 |
| 242467_at | *---* | *---* | 1.90 |
| 240221_at | *CSNK1A1* | Casein kinase 1, alpha 1 | 2.17 |
| 242191_at | *NBPF10* | neuroblastoma breakpoint family, member 10 | 1.80 |
| 213089_at | *LOC100132134* | similar to LOC653391 protein | 1.89 |
| 242403_at | *---* | *---* | 1.77 |
| 242749_at | *---* | *---* | 1.88 |
| 232890_at | *---* | *---* | 1.66 |
| 209210_s_at | *FERMT2* | fermitin family homolog 2 (Drosophila) | 1.63 |
| 215599_at | *SMA4* | glucuronidase, beta pseudogene | 2.05 |
| 238449_at | *LOC595101* | PI-3-kinase-related kinase SMG-1 pseudogene | 1.82 |
| 237746_at | *SFRS11* | Splicing factor, arginine/serine-rich 11 | 2.13 |
| 221773_at | *ELK3* | ELK3, ETS-domain protein (SRF accessory protein 2) | 1.50 |
| 224811_at | *---* | *---* | 1.58 |
| 235926_at | *---* | *---* | 1.70 |
| 230270_at | *PRPF38B* | PRP38 pre-mRNA processing factor 38 (yeast) domain containing B | 1.64 |
| 243410_at | *---* | *---* | 1.77 |
| 244778_x_at | *---* | *---* | 1.70 |
| 235716_at | *---* | *---* | 1.65 |
| 215888_at | *PDS5B* | PDS5, regulator of cohesion maintenance, homolog B (S. cerevisiae) | 1.78 |
| 226142_at | ***GLIPR1*** | GLI pathogenesis-related 1 | 1.62 |
| 240231_at | *---* | *---* | 2.02 |
| 235138_at | *---* | *---* | 1.70 |
| 236114_at | *---* | *---* | 1.67 |
| 229694_at | *BRWD2* | bromodomain and WD repeat domain containing 2 | 1.55 |
| 230712_at | *KIAA1245* | KIAA1245 | 1.77 |
| 215268_at | *KIAA0754* | hypothetical LOC643314 | 1.54 |
| 219025_at | *CD248* | CD248 molecule, endosialin | 1.54 |
| 226663_at | *ANKRD10* | ankyrin repeat domain 10 | 1.42 |
| 204774_at | *EVI2A* | ecotropic viral integration site 2A | 1.51 |
| 202270_at | *GBP1* | guanylate binding protein 1, interferon-inducible, 67kDa | 1.62 |
| 238156_at | *---* | *---* | 1.87 |
| 244165_at | *C10orf18* | chromosome 10 open reading frame 18 | 1.64 |
| 239243_at | *ZNF638* | Zinc finger protein 638 | 2.05 |
| 232521_at | *PCSK7* | Proproteinconvertasesubtilisin/kexin type 7 | 1.58 |
| 200878_at | *EPAS1* | endothelial PAS domain protein 1 | 1.57 |
| 236974_at | *---* | *---* | 1.60 |
| 240307_at | *---* | *---* | 1.46 |
| 226980_at | *DEPDC1B* | DEP domain containing 1B | 1.44 |
| 235646_at | *---* | *---* | 1.49 |
| 233445_at | *---* | *---* | 1.74 |
| 210164_at | *GZMB* | granzyme B (granzyme 2, cytotoxic T-lymphocyte-associated serine esterase 1) | 1.37 |
| 235595_at | *ARHGEF2* | Rho/rac guanine nucleotide exchange factor (GEF) 2 | 1.63 |
| 230493_at | *SHISA2* | shisa homolog 2 (Xenopus laevis) | 1.37 |
| 222266_at | *C19orf2* | Chromosome 19 open reading frame 2 | 1.40 |
| 230713_at | *---* | *---* | 1.51 |
| 209948_at | *KCNMB1* | potassium large conductance calcium-activated channel, subfamily M, beta member 1 | 1.57 |
| 239545_at | *---* | *---* | 1.62 |
| 228692_at | *---* | *---* | 1.42 |
| 214657_s_at | *TncRNA* | Trophoblast-derived noncoding RNA | 1.78 |
| 223381_at | *NUF2* | NUF2, NDC80 kinetochore complex component, homolog (S. cerevisiae) | 1.38 |
| 238142_at | *---* | *---* | 1.68 |
| 238988_at | *---* | *---* | 1.76 |
| 228723_at | *---* | *---* | 1.40 |
| 206584_at | *LY96* | lymphocyte antigen 96 | 1.38 |
| 241242_at | *---* | *---* | 1.36 |
| 239002_at | *ASPM* | asp (abnormal spindle) homolog, microcephaly associated (Drosophila) | 1.55 |
| 228180_at | *---* | *---* | 1.79 |
| 224875_at | *C5orf24* | chromosome 5 open reading frame 24 | 1.43 |
| 202949_s_at | *FHL2* | four and a half LIM domains 2 | 1.46 |
| 236462_at | *---* | *---* | 1.58 |
| 237561_x_at | *---* | *---* | 1.46 |
| 228729_at | *CCNB1* | cyclin B1 | 1.33 |
| 238311_at | *---* | *---* | 1.43 |
| 228087_at | *CCDC126* | coiled-coil domain containing 126 | 1.39 |
| 242060_x_at | *PHF11* | PHD finger protein 11 | 1.43 |
| 204491_at | *PDE4D* | phosphodiesterase 4D | 1.42 |
| 218376_s_at | *MICAL1* | microtubule associated monoxygenase, calponin and LIM domain containing 1 | 1.48 |
| 218646_at | *C4orf27* | chromosome 4 open reading frame 27 | 1.32 |
| 237107_at | *PRKRA* | protein kinase, interferon-inducible double stranded RNA dependent activator | 1.61 |
| 244219_at | *---* | *---* | 1.50 |
| 232356_at | *---* | *---* | 1.48 |
| 233678_at | *---* | *---* | 1.74 |
| 202932_at | *YES1* | v-yes-1 Yamaguchi sarcoma viral oncogene homolog 1 | 1.29 |
| 221985_at | *KLHL24* | kelch-like 24 (Drosophila) | 1.39 |
| 224799_at | *NDFIP2* | Nedd4 family interacting protein 2 | 1.30 |
| 236814_at | *MDM4* | Mdm4 p53 binding protein homolog (mouse) | 1.40 |
| 215012_at | *ZNF451* | zinc finger protein 451 | 1.49 |
| 200808_s_at | *ZYX* | zyxin | 1.33 |
| 239188_at | *PPP2R3C* | protein phosphatase 2 (formerly 2A), regulatory subunit B'', gamma | 1.35 |
| 208178_x_at | *TRIO* | triple functional domain (PTPRF interacting) | 1.28 |
| 226181_at | *TUBE1* | tubulin, epsilon 1 | 1.38 |
| 242310_at | *---* | *---* | 1.32 |
| 243388_at | *---* | *---* | 1.47 |
| 227198_at | *AFF3* | AF4/FMR2 family, member 3 | 1.58 |
| 204035_at | *SCG2* | secretogranin II (chromogranin C) | 1.46 |
| 226184_at | *FMNL2* | formin-like 2 | 1.32 |
| 224917_at | *MIRN21* | microRNA 21 | 1.55 |
| 217653_x_at | *---* | *---* | 1.39 |
| 236841_at | *LOC100133859* | hypothetical protein LOC100133859 | 1.34 |
| 211559_s_at | *CCNG2* | cyclin G2 | 1.28 |
| 204780_s_at | *FAS* | Fas (TNF receptor superfamily, member 6) | 1.25 |
| 225265_at | *RBMS1* | RNA binding motif, single stranded interacting protein 1 | 1.27 |
| 217989_at | *HSD17B11* | hydroxysteroid (17-beta) dehydrogenase 11 | 1.25 |
| 214918_at | *HNRNPM* | heterogeneous nuclear ribonucleoprotein M | 1.34 |
| 235028_at | *---* | *---* | 1.56 |
| 216202_s_at | *SPTLC2* | serine palmitoyltransferase, long chain base subunit 2 | 1.36 |
| 230489_at | *CD5* | CD5 molecule | 1.31 |
| 216060_s_at | *DAAM1* | dishevelled associated activator of morphogenesis 1 | 1.28 |
| 226665_at | *AHSA2* | AHA1, activator of heat shock 90kDa protein ATPase homolog 2 (yeast) | 1.36 |
| 212384_at | *BAT1* | HLA-B associated transcript 1 | 1.46 |
| 239151_at | *CTGLF6* | centaurin, gamma-like family, member 6 | 1.42 |
| 238119_at | *---* | *---* | 1.28 |
| 223966_at | *LOC100128180* | PRO2116 | 1.40 |
| 218762_at | *ZNF574* | zinc finger protein 574 | -1.41 |
| 210130_s_at | *TM7SF2* | transmembrane 7 superfamily member 2 | -1.48 |
| 209706_at | *NKX3-1* | NK3 homeobox 1 | -1.29 |
| 236196_at | *---* | *---* | -1.24 |
| 203761_at | *SLA* | Src-like-adaptor | -1.32 |
| 228049_x_at | *---* | *---* | -1.22 |
| 206727_at | *C9* | complement component 9 | -1.30 |
| 212186_at | *ACACA* | acetyl-Coenzyme A carboxylase alpha | -1.38 |
| 218897_at | *TMEM177* | transmembrane protein 177 | -1.48 |
| 240089_at | *---* | *---* | -1.27 |
| 209806_at | *HIST1H2BK* | histone cluster 1, H2bk | -1.49 |
| 237563_s_at | *LOC440731* | similar to hCG1817424 | -1.32 |
| 209372_x_at | *TUBB2A* | tubulin, beta 2A | -1.30 |
| 222040_at | *HNRNPA1* | heterogeneous nuclear ribonucleoprotein A1 | -1.28 |
| 223839_s_at | *SCD* | stearoyl-CoA desaturase (delta-9-desaturase) | -1.40 |
| 201502_s_at | *NFKBIA* | nuclear factor of kappa light polypeptide gene enhancer in B-cells inhibitor, alpha | -1.37 |
| 209608_s_at | *ACAT2* | acetyl-Coenzyme A acetyltransferase 2 | -1.36 |
| 231088_at | *LOC340544* | Hypothetical protein LOC340544 | -1.30 |
| 208647_at | *FDFT1* | farnesyl-diphosphate farnesyltransferase 1 | -1.29 |
| 239848_at | *---* | *---* | -1.46 |
| 224185_at | *---* | *---* | -1.52 |
| 227778_at | *KIAA1833* | hypothetical protein KIAA1833 | -1.46 |
| 223707_at | *RPL27A* | ribosomal protein L27a | -1.42 |
| 212218_s_at | *FASN* | fatty acid synthase | -1.47 |
| 230944_at | *---* | *---* | -1.45 |
| 222449_at | *PMEPA1* | prostate transmembrane protein, androgen induced 1 | -1.43 |
| 202245_at | *LSS* | lanosterol synthase (2,3-oxidosqualene-lanosterol cyclase) | -1.66 |
| 225826_at | *MMAB* | methylmalonicaciduria (cobalamin deficiency) cblB type | -1.57 |
| 212242_at | *TUBA4A* | tubulin, alpha 4a | -1.53 |
| 210010_s_at | *SLC25A1* | solute carrier family 25 (mitochondrial carrier; citrate transporter), member 1 | -1.65 |
| 213787_s_at | *EBP* | emopamil binding protein (sterol isomerase) | -1.50 |
| 213577_at | *SQLE* | squalene epoxidase | -1.58 |
| 240546_at | *LOC389043* | hypothetical gene supported by AK125982; BC042817 | -1.56 |
| 221750_at | *HMGCS1* | 3-hydroxy-3-methylglutaryl-Coenzyme A synthase 1 (soluble) | -1.88 |
| 238725_at | *IRF1* | interferon regulatory factor 1 | -1.63 |
| 202562_s_at | *C14orf1* | chromosome 14 open reading frame 1 | -1.51 |
| 228854_at | *---* | *---* | -1.80 |
| 227044_at | *---* | *---* | -1.69 |
| 200862_at | *DHCR24* | 24-dehydrocholesterol reductase | -1.83 |
| 205898_at | ***CX3CR1*** | chemokine (C-X3-C motif) receptor 1 | -2.25 |
| 201790_s_at | *DHCR7* | 7-dehydrocholesterol reductase | -1.95 |
